# Si-Ni-San alleviates depression-like behavior via regulating the gut microbiota-tryptophan metabolism-AhR/NF-κB pathway axis

**DOI:** 10.1186/s13020-026-01390-4

**Published:** 2026-04-07

**Authors:** Qiang Xiao, Zhaoyi Wen, Huang Zhan, Han Zhao, Yukun Jiao, Dehua Huang, Hui Li, Congcong Chen

**Affiliations:** 1 Jiangxi Province Key Laboratory of Traditional Chinese Medicine Pharmacology, Institute of Traditional Chinese Medicine Health Industry, China Academy of Chinese Medical Sciences, Nanchang, 330115 China; 2 Jiangxi Health Industry Institute of Traditional Chinese Medicine, Nanchang, 330115 China; 3 Modern Research Center for Traditional Chinese Medicine, The Key Laboratory of Chemical Biology and Molecular Engineering of Ministry of Education, Shanxi University, Taiyuan, 030006 China; 4 Institute of Chinese Materia Medica, China Academy of Chinese Medical Sciences, Beijing, 100700 China

**Keywords:** Si-Ni-San, Gut microbiota, Tryptophan metabolism, Indole-3-acetic acid, AhR/NF-κB, Depression

## Abstract

**Background:**

Si-Ni-San (SNS), a classic herbal formula from the *Treatise on Cold Damage Diseases*, is used to treat depression by relieving “liver qi stagnation”. However, the underlying mechanism remains unclear.

**Purpose of the research:**

This study aimed to investigate the mechanism by which SNS alleviates depression-like behavior, specifically focusing on its role in modulating gut microbiota and host tryptophan metabolism.

**Methods:**

A depression model was induced in mice by chronic unpredictable mild stress (CUMS). The antidepressant effects of SNS were evaluated through behavioral tests. Integrated untargeted and targeted metabolomics, alongside 16S rRNA sequencing, were utilized to identify potential gut-brain signaling molecules. Molecular interactions between the gut-brain signaling molecule and its target were validated by surface plasmon resonance (SPR) and molecular docking. Key protein expression was measured via Western blot and ELISA. Finally, the function of gut microbiome-derived indole-3-acetic acid (IAA) as a key gut-brain signaling molecule was confirmed by oral supplementation experiments.

**Results:**

SNS significantly alleviated CUMS-induced depression-like behaviors. Multi-omics analysis revealed that SNS reversed tryptophan metabolic disorders and elevated gut microbiome-derived IAA levels in both the colon and prefrontal cortex, which was attributed to the enrichment of *Lactobacillus*. Further investigations confirmed that IAA directly binds to and activates the aryl hydrocarbon receptor (AhR), thereby inhibiting NF-κB pathway-mediated neuroinflammation. Moreover, oral supplementation with IAA replicated the antidepressant effects of SNS and suppressed CUMS-induced neuroinflammation via the AhR/NF-κB signaling pathway.

**Conclusion:**

SNS alleviates depression-like behavior by modulating gut microbiota-mediated tryptophan metabolism to enhance IAA production, thereby activating central AhR signaling and suppressing NF-κB-mediated neuroinflammation.

**Graphical Abstract:**

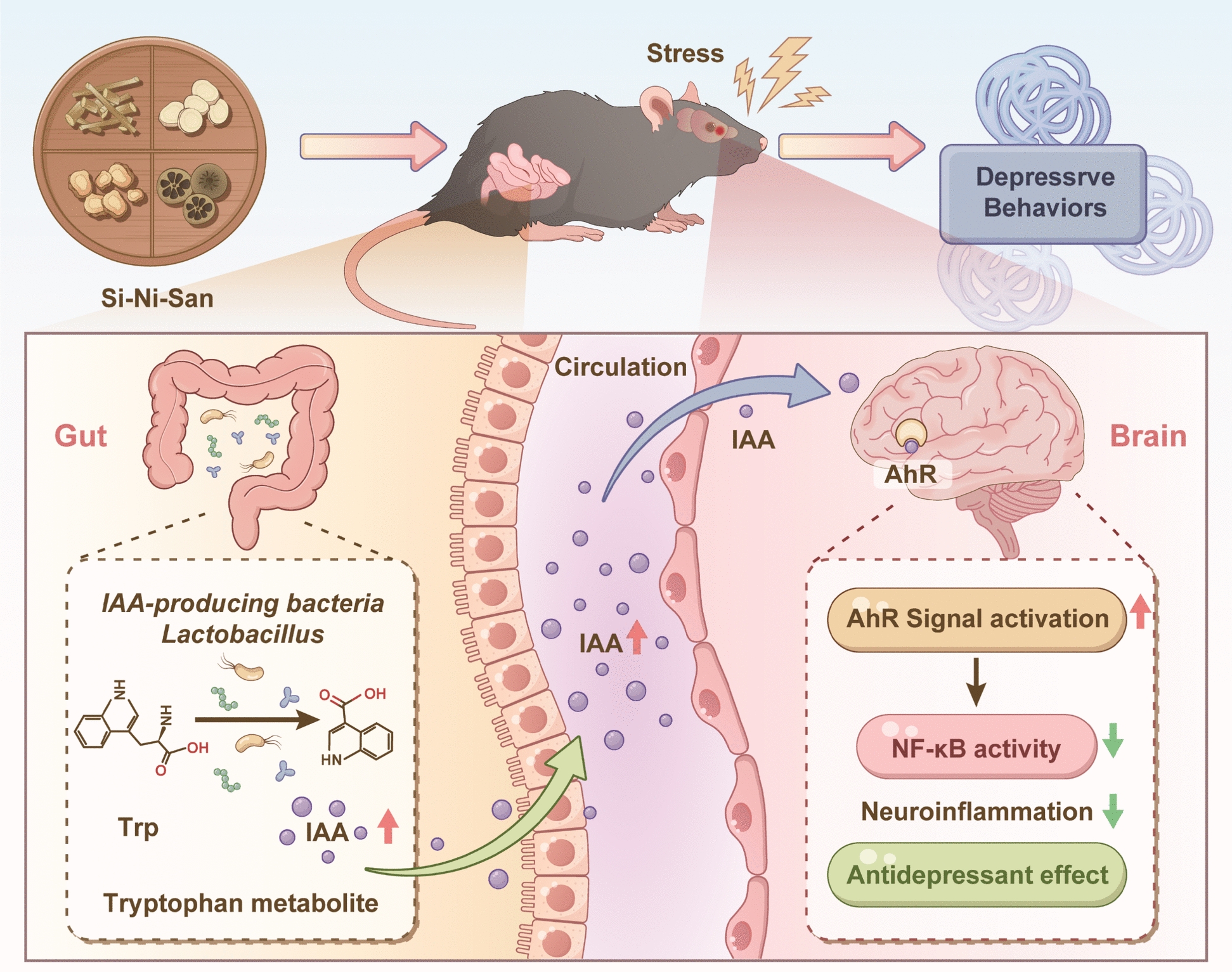

**Supplementary Information:**

The online version contains supplementary material available at 10.1186/s13020-026-01390-4.

## Introduction

Major Depressive Disorder is a debilitating global public health issue, affecting more than 280 million people worldwide and standing as a leading contributor to disability and suicide-related deaths. The development of effective treatments has been substantially impeded because the disorder’s pathophysiology is yet to be fully elucidated, despite considerable research efforts [1, 2]. The classical monoaminergic hypothesis—which emphasizes deficits in serotonin, noradrenaline, and dopamine neurotransmission—has long guided antidepressant development. Yet, this framework fails to explain the delayed therapeutic onset of conventional antidepressants like SSRIs, nor does it fully account for the high rate of treatment resistance, affecting nearly one-third of patients [3, 4]. These limitations have prompted the investigation of alternative pathophysiological mechanisms. In this context, the gut–brain axis has gained prominence as a key area for elucidating and addressing depression. This system comprises a dynamic, bidirectional communication network linking the gut to the central nervous system. This interaction is facilitated via neural, immunological, and metabolic pathways and is significantly influenced by the gut microbiota. This complex intestinal ecosystem produces a diverse array of bioactive metabolites that can profoundly modulate host physiology and behavior [5]. Growing clinical and preclinical evidence consistently reveals gut dysbiosis—defined as alterations in microbial composition, diversity, and metabolic activity—in individuals with depression [6]. Importantly, fecal microbiota transplantation (FMT) using donors with depression or from stressed animals can induce depression-related behavioral phenotypes in healthy recipients, demonstrating the causal involvement of gut microbes in this disorder [7]. Therefore, targeting the gut microbiota to reestablish homeostasis along the microbiota–gut–brain axis represents an emerging therapeutic opportunity for major depressive disorder.

Within the gut–brain axis, tryptophan metabolism represents a pivotal link connecting gut microbial activity to central nervous system function. As an essential amino acid, tryptophan fulfills two fundamental roles: it acts as the exclusive precursor in the biosynthesis of cerebral serotonin—a key neurotransmitter in mood regulation—and is metabolized by gut microbes into a spectrum of bioactive compounds, notably indole-3-acetic acid and indole-3-aldehyde [8, 9]. Unlike host-derived metabolites, these microbially produced indoles act as endogenous aryl hydrocarbon receptor (AhR) ligands. AhR is a ligand-activated transcription factor that serves as a molecular conduit, transducing gut-derived microbial signals to the brain. It is vital for immune homeostasis, and its activation mediates potent anti-inflammatory actions [10]. Widely expressed in immune cells, neurons, and microglia, AhR modulates immunity by suppressing pro-inflammatory cascades. In the central nervous system, AhR activation directly inhibits nuclear factor-κB signaling, a pathway that is frequently hyperactivated in depression and drives neuroinflammation [11]. This inhibition suppresses the production and secretion of key pro-inflammatory mediators, notably TNF-α and IL-6, thereby alleviating glia-mediated neuronal injury [12]. Through these mechanisms, AhR serves as a key molecular regulator in counteracting neuroinflammation, a central pathological process in depression [13].

Si-Ni-San (SNS) is a classic herbal formula originating from the “Treatise on Cold Damage Diseases” compiled by Zhang Zhongjing in the Eastern Han Dynasty. The formula consists of four medicinal components: Bupleuri Radix (*Bupleurum chinense* DC), Paeoniae Radix Alba (*Paeonia lactiflora* Pall), Aurantii Fructus Immaturus (*Citrus aurantium* L), and Glycyrrhizae Radix et Rhizoma (*Glycyrrhiza glabra* L). In traditional Chinese medicine (TCM), SNS is historically indicated for the “Yang Depression and Cold Extremities” syndrome, which is characterized by emotional constraint and stagnation of Qi—symptoms that correspond closely to the core manifestations of depressive disorders [14]. The modern application of SNS in depression is grounded in the TCM concept of “Qi stagnation,” a well-established pathological mechanism underlying emotional dysregulation. Accordingly, contemporary TCM practice has systematically adopted SNS as a foundational formula for managing depression, reflecting a direct continuity from its traditional use, as documented in both clinical observations and theoretical studies [15–18]. This traditional indication is further supported by recent pharmacological research and controlled clinical trials that confirm the antidepressant effects of SNS [19–21]. Evidence from diverse animal studies indicates its effectiveness in reducing depressive-like symptoms via multiple pathways [22–24]. These include elevating central 5-hydroxytryptamine levels, suppressing hypothalamic–pituitary–adrenal axis hyperactivity, and reducing neuroinflammation [25]. Recent research has further demonstrated that SNS enhances mitochondrial performance and reinstates synaptic plasticity in models of maternal separation. Additionally, it repairs synaptic injury within the dorsal raphe nucleus via calcium-sensing receptor signaling, and modulates neural circuit connectivity between the prefrontal cortex and the dorsal raphe nucleus [17, 26, 27]. Moreover, SNS strengthens the intestinal barrier, regulates systemic fat absorption, and modulates ferritinophagy through NCOA4, influencing dendritic spine remodeling [28]. Although considerable progress has been made, whether SNS alleviates depression via the gut–brain axis, especially through regulation of intestinal flora and their metabolic products, has not been fully established. This uncertainty highlights the need to investigate how microbiota-derived metabolites mediate central SNS actions.

Although earlier research has indicated the antidepressant potential of SNS and its capacity to modulate gut microbiota, the specific signaling metabolites mediating gut–brain communication and their associated molecular pathways remain poorly understood. Here, via integrated metabolomic and 16S rRNA sequencing analyses, we observed a marked decrease in gut microbiome-derived indole-3-acetic acid (IAA) in both the colon and prefrontal cortex of CUMS-induced depressed mice. Building on this observation, we examined whether SNS alleviates depression via regulation of the gut microbiota–tryptophan metabolism-IAA axis. We further elucidated the function of IAA as a gut–brain signaling metabolite that, upon reaching the prefrontal cortex, activates AhR and subsequently inhibits NF-κB-driven neuroinflammation. Our results indicate that SNS mitigates depression-like behavior by enriching IAA-producing Lactobacillus, raising central IAA levels, and activating the AhR/NF-κB pathway, thus offering a mechanistic explanation for the gut microbiota–dependent antidepressant effect of SNS.

## Materials and methods

### Materials and reagents

The four constituent herbs of SNS—Radix Bupleuri (No. 2403001), Radix Paeoniae Alba (No. 2403002), Fructus Aurantii Immaturus (No. 2403003), and Radix et Rhizoma Glycyrrhizae (No. 2403004)—were supplied by Anguo Changda Chinese Herbal Medicine Pieces Co., Ltd. (Anguo, China) and met the quality standards stipulated in the Chinese Pharmacopoeia (2025). All these herbs are preserved in the herbarium of the Institute of Traditional Chinese Medicine Health Industry, China Academy of Chinese Medical Sciences. Indole-3-acetic acid (IAA) was sourced from Sigma-Aldrich (St. Louis, MO, USA). Recombinant human aryl hydrocarbon receptor (AhR) and antibodies targeting phosphorylated NF-κB (*p*-p65, cat. no. ab86299), total NF-κB (p65, cat. no. ab32536), AhR (cat. no. ab190797), and *β*-actin (cat. no. ab6276) were supplied by Abcam (Cambridge, UK).

### Animals

Male C57BL/6 J mice were acquired from Beijing Sibeifu Biotechnology Co., Ltd. (Beijing, China). The animals were maintained in a regulated environment with a 12/12-h light–dark cycle, a temperature range of 23–25 °C, and 50–65% relative humidity. After a 7-day acclimatization period, all experimental steps were implemented. The study protocol received ethical approval from the Animal Ethics Committee of the Institute of Traditional Chinese Medicine Health Industry, China Academy of Chinese Medical Sciences (approval number: 2024022), and was carried out following the National Institutes of Health guidelines for the care and use of laboratory animals.

### Chronic unpredictable mild stress (CUMS) procedure

Following a previously described protocol [29] with minor adjustments, CUMS was implemented in this study. In short, after the acclimatization phase, animals underwent daily exposure to a series of randomized mild stressors over a period of 28 days. The stress regimen included: (1) 5 min forced swim in 4 °C water; (2) intermittent foot shock (36 V, 2 min); (3) tail clamping for 2 min; (4) exposure to 60 dB noise for 3 h; (5) 12 h light/dark cycle reversal; (6) 10 min heat stress at 45 °C; (7) physical restraint for 2 h; (8) 24 h food deprivation; and (9) 24 h water deprivation. Stressors were applied in a variable sequence to prevent habituation.

### Preparation of SNS extract

The aqueous extract of SNS was prepared according to established methods [17, 26]. Briefly, the four component herbs—Radix Bupleuri, Radix Paeoniae Alba, Fructus Aurantii Immaturus, and Radix et Rhizoma Glycyrrhizae—were weighed in equal proportions (1:1:1:1) and immersed in distilled water (1:10, *w/v*) for 2 h. The mixture was subsequently reflux-extracted twice using the same volume of water, each extraction lasting 2 h. The resulting solutions were combined, concentrated under reduced pressure, and lyophilized to yield a powdered extract, with a final yield of 11.12%. Quality control of the SNS extract was performed using HPLC fingerprinting. Paeoniflorin, liquiritin, naringin, and hesperidin were used as reference markers for quality assessment. Detailed chromatographic conditions are provided in Supplementary File 1, and a representative HPLC chromatogram is shown in Fig. S1. The RSDs of the relative retention times were less than 1.0%, and those of the relative peak areas were below 3.0% for all common peaks. By comparison with chemical reference substances, the main peaks in SNS were identified as paeoniflorin (tᵣ = 4.928 min), liquiritin (tᵣ = 6.005 min), naringin (tᵣ = 8.075 min), and hesperidin (tᵣ = 8.819 min), confirming the consistency and quality of the SNS extract used in this study.

### Drug administration

After a 7-day acclimatization period, the mice were randomly assigned into six distinct groups, with n = 10 in each. The groups comprised a control (CON), a CUMS model (CUMS), groups receiving SNS at low, medium, or high doses (designated SNS-L, SNS-M, and SNS-H), and a positive control group administered fluoxetine (FLX). The SNS doses were set at 1.8, 3.6, and 7.2 g herb/kg, and fluoxetine was administered at 5 mg/kg, based on previously effective doses [17, 26]. The lyophilized SNS powder and fluoxetine were dissolved in 0.9% saline for intragastric administration. The corresponding SNS-L, SNS-M, and SNS-H groups received 200, 400, and 800 mg of lyophilized powder per kg body weight, respectively, while the FLX group received 5 mg/kg fluoxetine. All treatments were delivered daily at 10 mL/kg body weight. Both the CUMS and drug-treated groups were subjected to the 28-day CUMS protocol, during which drug treatments were conducted concurrently. All mice were fasted for 12 h prior to being anesthetized via intraperitoneal injection of pentobarbital sodium (30 mg/kg). Subsequently, euthanasia was performed by cervical dislocation. Throughout the study, no animals exhibited abnormal clinical signs or met predefined humane endpoint criteria.

### Behavioral assessments

The antidepressant-like properties of SNS were assessed using the sucrose preference test (SPT), tail suspension test (TST), forced swim test (FST), and open field test (OFT), following established protocols [30]. Detailed procedures for each behavioral assay are provided in Supplementary File 2.

### Untargeted metabolomic profiling

Metabolite extraction and preparation from prefrontal cortex tissue were conducted following our established protocol [30], with comprehensive details available in Supplemental File 3. Untargeted metabolomic data acquisition was performed using a Thermo Fisher Dionex UltiMate 3000 UHPLC system coupled to a Q Exactive Orbitrap mass spectrometer (UHPLC-Q-Orbitrap/MS), controlled by Xcalibur software (Thermo Fisher, MA, USA). The specific chromatographic and mass spectrometric parameters are provided in Supplemental File 4.

The raw data were initially processed with Compound Discoverer 3.0 (Thermo Fisher, MA, USA) to align peaks, identify compounds, and perform quantification, following the procedure detailed in Supplemental File 5. Subsequent multivariate analyses, namely principal component analysis (PCA) and orthogonal partial least squares discriminant analysis (OPLS-DA), were performed in SIMCA 16.0 (Umetrics, Sweden). Metabolites were deemed statistically significant if they satisfied the following criteria: variable importance in projection (VIP) > 1.0, −log10(*P*) > 1.3 (*P* < 0.05), and an absolute log2 fold-change |log2(FC)|> 1.0. Confirmation of metabolite identities was achieved by matching accurate mass, MS/MS spectra, and retention times against authentic standards and by querying the HMDB, KEGG, Lipid Maps, and Metlin databases.

### Targeted metabolomic analysis

Concentrations of indole-3-acetic acid (IAA) in the prefrontal cortex and colon content samples were quantified using a targeted metabolomics approach, as previously described [30] (see Supplemental File 6 for details). Analyses were conducted using an Agilent 1290 UHPLC system (Agilent Technologies, USA) interfaced with a Q-TRAP 6500 + mass spectrometer (AB Sciex, USA). Analysis was performed using dynamic multiple reaction monitoring (MRM) on a mass spectrometer, with separation conducted on an Acquity UPLC HSS T3 column (100 mm × 2.1 mm, 1.8 μm; Waters, USA). Optimized MRM conditions for each compound, such as transition pairs, collision energy (CE), and declustering potential (DP), are provided in Supplementary Table S1. Detailed UHPLC and MS conditions are provided in Supplemental File 6.

### Gut microbiota depletion experiment

To determine whether the antidepressant effects of SNS are mediated by the gut microbiota, a separate experiment involving gut microbiota depletion was conducted. Following a 7-day acclimatization period, mice were randomly allocated into four groups (n = 8 per group): control (CON), CUMS model (CUMS), SNS treatment (SNS, 7.2 g herb/kg, equivalent to 800 mg lyophilized powder/kg), and SNS combined with antibiotic treatment (SNS + ABX). For broad-spectrum antibiotic treatment, mice in the SNS + ABX group received a mixture of vancomycin (0.5 mg/mL), gentamicin (1 mg/mL), ampicillin (0.5 mg/mL), and streptomycin (1 mg/mL) in their drinking water ad libitum throughout the entire 28-day CUMS protocol to deplete the gut microbiota. The lyophilized SNS powder was dissolved in 0.9% saline and administered intragastrically once daily at a volume of 10 mL/kg body weight. Concurrently with the 28-day CUMS procedure, mice in the SNS and SNS + ABX groups received SNS treatment, while the CON and CUMS groups received an equivalent volume of vehicle. Following the behavioral assessments, all mice were fasted for 12 h prior to being anesthetized via intraperitoneal injection of pentobarbital sodium (30 mg/kg). Subsequently, euthanasia was performed by cervical dislocation. Throughout the study, no animals exhibited abnormal clinical signs or met predefined humane endpoint criteria.

### 16S rRNA gene sequencing and microbiota analysis

Genomic DNA was isolated from fresh colon content using the cetyltrimethylammonium bromide (CTAB) method according to the provided protocol. Amplification of the bacterial 16S rRNA gene hypervariable regions was performed with universal primers. The amplified products were then analyzed on a 2% agarose gel and subsequently sequenced on an Illumina platform at LC-Bio Technology Co., Ltd. (Hangzhou, China). Sequencing reads were processed and analyzed to assess microbial diversity and composition. Alpha diversity, reflecting within-sample richness and evenness, was evaluated using the Chao1 index (species richness) and the Shannon index (community diversity). Beta diversity, representing compositional differences between samples, was visualized via principal component analysis (PCA) and principal coordinates analysis (PCoA). Taxonomic composition at the genus level was summarized and displayed using stacked bar charts.

### Western blot analysis

Protein levels of the aryl hydrocarbon receptor (AhR), phosphorylated NF-κB (*p*-p65), and total NF-κB (p65) in the prefrontal cortex were assessed via western blotting. Following the manufacturer’s protocol, prefrontal cortex tissues were homogenized using RIPA lysis buffer. A bicinchoninic acid (BCA) assay kit (Nanjing Jiancheng Bioengineering Institute, China) was employed to measure protein concentrations. Protein samples were heat-denatured at 100 °C for 5 min in loading buffer. Subsequently, equivalent protein quantities from all samples were resolved via SDS-PAGE and electrophoretically transferred to polyvinylidene fluoride (PVDF) membranes. Following transfer, the membranes were subjected to a 1-h blocking step using 5% bovine serum albumin (BSA) dissolved in TBST (Tris-buffered saline containing 0.1% Tween 20). Thereafter, the membranes were incubated with the following specific primary antibodies at 4 °C overnight: anti-AhR (1:1000 dilution), anti-*p*-p65 (1:1500), anti-p65 (1:1500), and anti-*β*-actin (1:1500). After washing three times with TBST, the membranes were subsequently incubated with horseradish peroxidase (HRP)-linked secondary antibodies (1:10,000) for 2 h at room temperature. An enhanced chemiluminescence (ECL) substrate (Servicebio) was used to visualize the immunoreactive bands, and their intensities were quantified using ImageJ (version 1.8.0), normalized to *β*-actin as the loading control.

### Enzyme-linked immunosorbent assay (ELISA)

Levels of the pro-inflammatory cytokines IL-1*β*, IL-6, and TNF-*α* in prefrontal cortex homogenates were quantified with commercially available ELISA kits (Nanjing Jiancheng Bioengineering Institute, China). All steps were performed according to the manufacturer's protocols. The absorbance was read at the appropriate wavelength, and cytokine concentrations were calculated based on the standard curves.

### Surface plasmon resonance (SPR) assay

Molecular interaction between IAA and the AhR was assessed by surface plasmon resonance (SPR) using a Biacore^™^ 8 K system (Cytiva, USA). The recombinant human AhR protein was prepared at 10 µg/mL in PBS (pH 7.4) supplemented with 1% trehalose and 1% DMSO. It was then immobilized onto a CM5 sensor chip through amine coupling. The binding affinity of IAA for the immobilized AhR was evaluated using a single-cycle kinetics procedure. Sensorgrams were analyzed to determine the kinetic parameters.

### Molecular docking

The three-dimensional crystal structure of the AhR (PDB ID: 8XSA) was retrieved from the Protein Data Bank. Using AutoDock Tools 4.2.6, the protein structure was prepared for docking by adding hydrogen atoms and assigning Gasteiger partial charges. The molecular structure of IAA was downloaded from the ZINC database. The interaction between IAA (ligand) and AhR (receptor) was simulated using the CDOCKER protocol within Discovery Studio 2016 to predict the binding affinity and mode.

### IAA supplementation treatment

To further validate the role of IAA, a separate supplementation experiment was conducted. Following acclimatization, mice were randomly allocated into five groups (*n* = 10 per group): control (CON), CUMS model (CUMS), low-dose IAA (IAA-L, 30 mg/kg), high-dose IAA (IAA-H, 60 mg/kg), and a positive control group treated with SNS (SNS, 7.2 g herb/kg, equivalent to 800 mg lyophilized powder/kg). Both IAA and the SNS lyophilized powder were prepared in 0.9% saline. Each treatment was delivered intragastrically once daily at 10 mL/kg. Concurrently with the 28-day CUMS protocol, the mice in the corresponding groups received their respective drug treatments. All mice were fasted for 12 h prior to being anesthetized via intraperitoneal injection of pentobarbital sodium (30 mg/kg). Subsequently, euthanasia was performed by cervical dislocation. Throughout the study, no animals exhibited abnormal clinical signs or met predefined humane endpoint criteria.

### AhR antagonist rescue experiment

To further establish a causal link between AhR activation and the antidepressant effects of SNS and IAA, a pharmacological rescue experiment was conducted using the specific AhR inhibitor (AhRi), Stemregenin 1. Following a 7-day acclimatization period, mice were randomly allocated into five groups (*n* = 8 per group): control (CON), CUMS model (CUMS), CUMS + AhRi, CUMS + AhRi + SNS, and CUMS + AhRi + IAA. The CUMS protocol was applied to all groups except the CON group for 28 consecutive days. Mice in the AhRi, AhRi + SNS, and AhRi + IAA groups received daily intraperitoneal injections of Stemregenin 1 (30 mg/kg; MedChemExpress) throughout the 28-day CUMS period. Concurrently, the AhRi + SNS group was administered SNS (7.2 g herb/kg) by oral gavage, and the AhRi + IAA group received IAA (60 mg/kg) by oral gavage, once daily. The CON and CUMS groups received equivalent volumes of vehicle (0.9% saline) via both intraperitoneal injection and oral gavage to control for procedural effects. Following the final drug administration, all mice underwent a series of behavioral tests to assess depression-like behaviors. Subsequently, mice were fasted for 12 h prior to being anesthetized via intraperitoneal injection of pentobarbital sodium (30 mg/kg), and euthanasia was performed by cervical dislocation. Throughout the experiment, no animals exhibited abnormal clinical signs or met predefined humane endpoint criteria.

### Statistical analysis

Statistical outcomes are reported as mean ± standard deviation. Analyses were conducted with GraphPad Prism (ver. 8.2.1, USA). Prior to conducting analyses, the normality of the datasets was examined. Two independent groups were compared using either an unpaired two-tailed Student’s t-test or the Mann–Whitney test, based on data distribution characteristics. For comparisons involving multiple groups, one-way ANOVA was employed, with Tukey’s or Dunnett’s test applied for post hoc analysis. A threshold of *P* < 0.05 defined statistical significance.

## Results

### SNS exerted antidepressant-like activity in a mouse model of chronic unpredictable mild stress

**Fig. 1:**
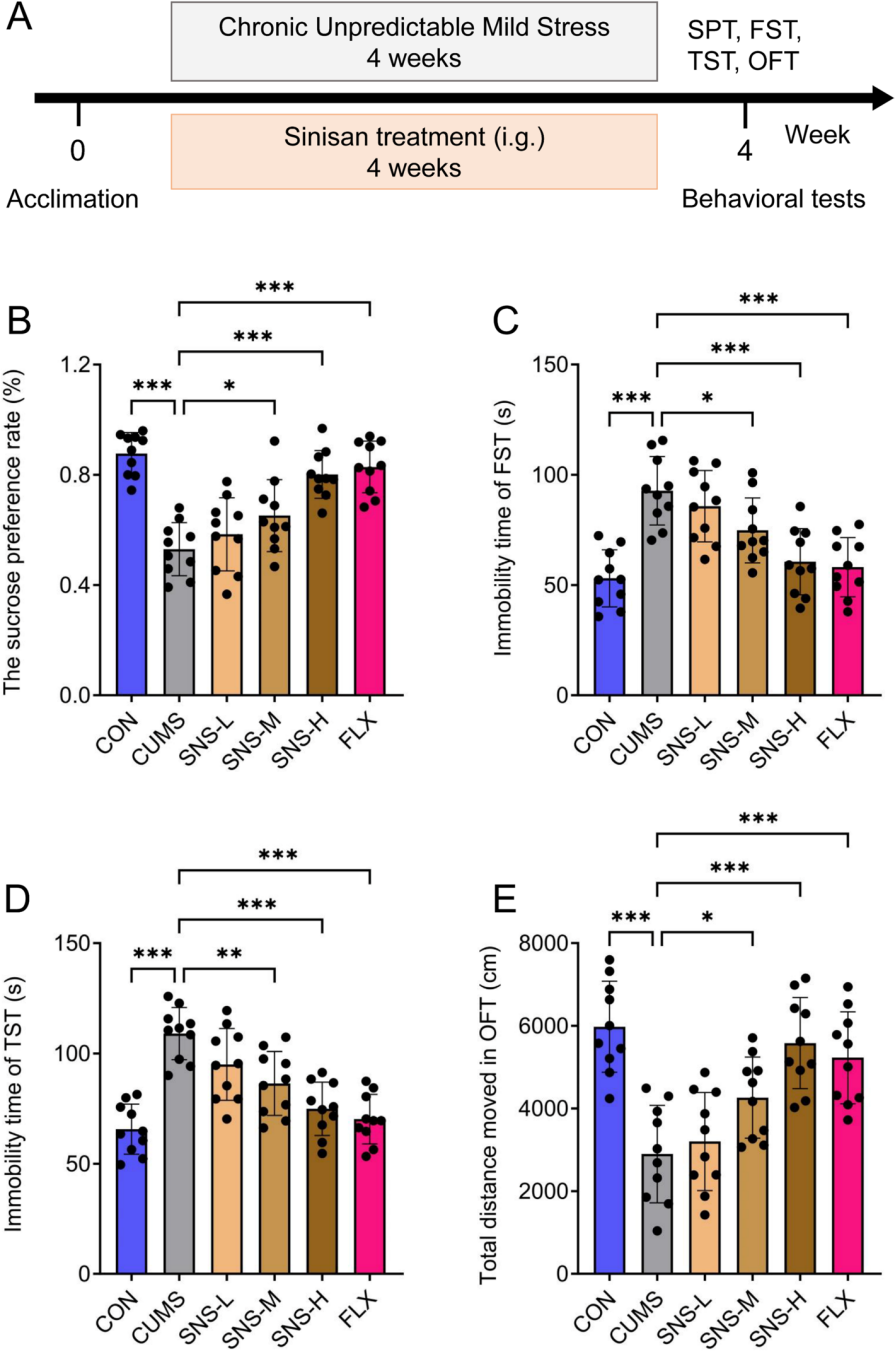
The antidepressant efficacy of SNS was evaluated in depressive mice subjected to CUMS. **A** Timeline of experimental procedures. **B** Sucrose preference test (SPT) outcomes for the control (CON), CUMS, SNS-treated (low, medium, high dose: SNS-L, SNS-M, SNS-H), and fluoxetine (FLX) groups. **C** Immobility time recorded in the FST. **D** Immobility time recorded in the TST. **E** Total movement distance in the OFT. All results are shown as mean ± SD (*n* = 10). *P* values were computed via two-tailed Student’s t-test (between two groups) or one-way ANOVA (among several groups). ^*^P < 0.05, ^**^P < 0.01, and ^***^P < 0.001, vs CUMS group

To investigate the efficacy and mechanism of SNS in treating depression, a depression mouse model was generated using the CUMS protocol. (Fig. 1A). Behavioral tests, including the SPT, TST, FST, OFT, revealed that compared with the CON group, the CUMS group exhibited a significant decrease in the sucrose preference rate and total distance moved in the OFT, along with a marked increase in immobility time in both the FST and TST, indicating the successful induction of depression-like behaviors (Fig. 1B–E). Treatment with SNS dose-dependently ameliorated these CUMS-induced depressive behaviors. Specifically, compared with the CUMS group, both the middle- and high-dose SNS groups showed a significant increase in sucrose preference rate and total distance moved in the OFT, as well as a significant reduction in immobility time in the FST and TST. Notably, the antidepressant effect of high-dose SNS was most pronounced and comparable to that of the positive control FLX (Fig. 1B–E). These results demonstrate that SNS treatment significantly reverses depression-like behaviors induced by CUMS in mice.

### The antidepressant effect of SNS is associated with the regulation of the tryptophan metabolism pathway in the prefrontal cortex

**Fig. 2:**
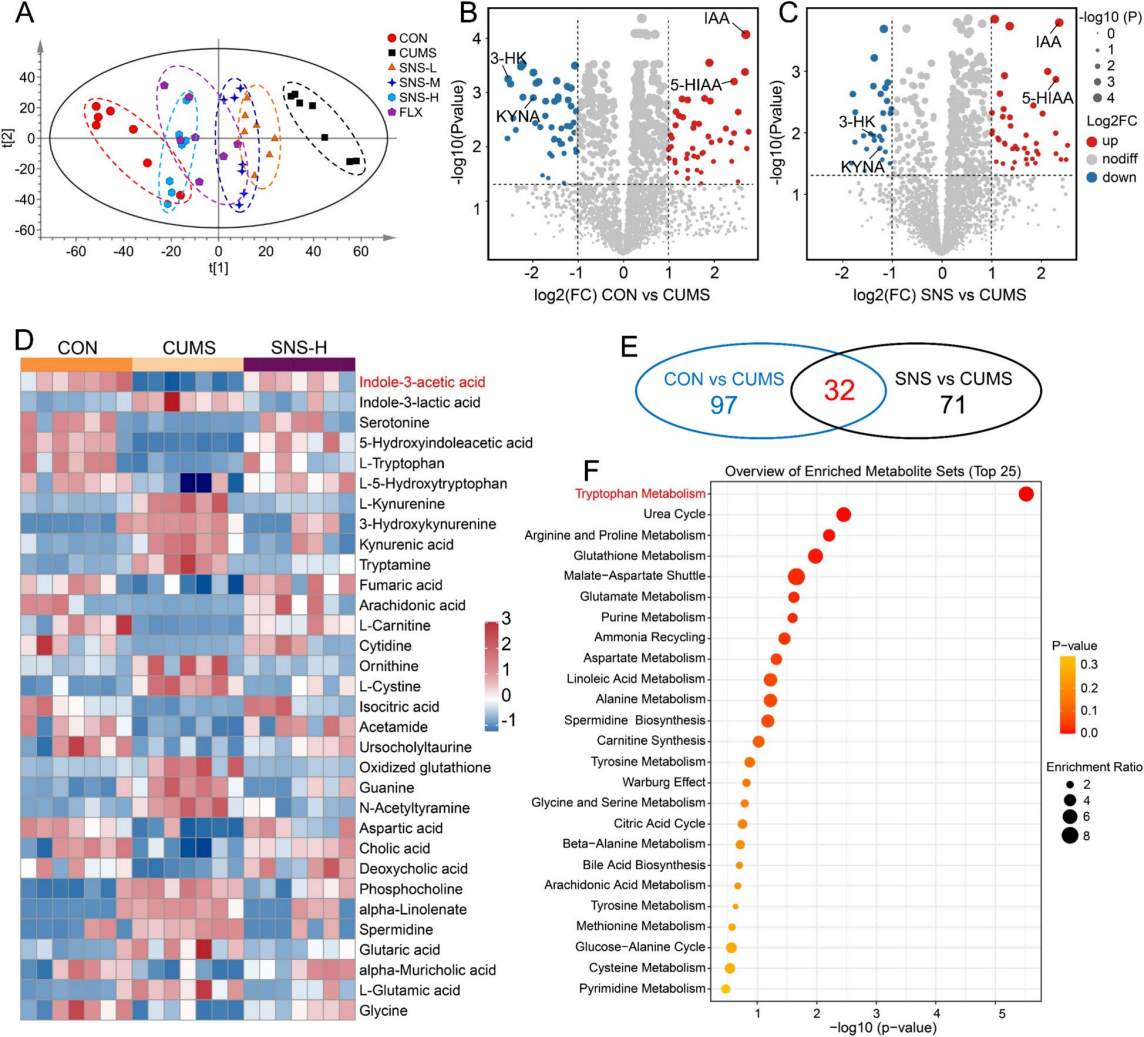
The antidepressant effect of SNS is mediated by the modulation of the tryptophan metabolism pathway in the prefrontal cortex. **A** Principal components analysis (PCA) score plots derived from untargeted metabolomic analyses of prefrontal cortex tissue samples (*n* = 7). **B** Volcano plot of untargeted metabolomic analyses of prefrontal cortex tissue samples from CON vs. CUMS group. **C** Volcano plot of untargeted metabolomic analyses of prefrontal cortex tissue samples from the SNS vs. CUMS group. **D** Clustering heatmap of the 32 overlapping differential metabolites identified across the CON vs. CUMS and SNS vs. CUMS comparisons. The color scale from red to blue depicts metabolite levels above or below the mean, respectively. **E** Venn diagram analysis illustrating the overlap of significant differential metabolites between the CON vs. CUMS and SNS vs. CUMS comparisons. **F** The pathway enrichment analysis of the 32 overlapping differential metabolites using MetaboAnalyst 6.0

To uncover the molecular pathways involved in the antidepressant efficacy of SNS, untargeted and targeted metabolomics were applied to prefrontal cortex tissue samples collected from mice in each group. The PCA and PLS-DA score plots generated by untargeted metabolomics showed a clear separation between the CUMS and CON groups. Notably, the metabolic profiles of the SNS-H and FLX groups were closer to the CON group and distinctly separated from the CUMS group, indicating that drug treatment significantly reversed the CUMS-induced metabolic disturbances in the prefrontal cortex (Fig. 2A and Supplementary Fig. S2). Furthermore, the metabolic profile of the SNS-H group more closely resembled that of the CON group than did the FLX group, suggesting that SNS-H may exert a more comprehensive restorative effect on the CUMS-induced metabolic alterations compared to FLX.

Subsequently, differential metabolites between the CON and CUMS groups, as well as between the SNS-H and CUMS groups, were screened based on a variable importance in projection (VIP) value > 1.0, a −log10(*P*) > 1.3 (equivalent to *P* < 0.05), and an absolute log2 fold-change |log2(FC)|> 1.0. This analysis identified 97 and 71 differential metabolites in the CON vs. CUMS and SNS-H vs. CUMS comparisons, respectively (Fig. 2B, C). Among these, the tryptophan metabolism metabolite IAA was markedly decreased in the CUMS group, and this reduction was significantly reversed by SNS-H treatment (Fig. 2D). Further analysis identified 32 overlapping differential metabolites between the CON vs. CUMS and SNS-H vs. CUMS comparisons (Fig. 2E). Pathway analysis of these 32 metabolites using MetaboAnalyst 6.0 demonstrated that the tryptophan metabolism pathway was the most significantly impacted pathway in both the metabolic pathway analysis and the pathway enrichment analysis (Fig. 2F and Supplementary Fig. S3). It is worth noting that the tryptophan metabolism pathway is co-metabolized by the host and the gut microbiota, generating a variety of signaling metabolites that have been shown to be versatile modulators of gut-brain axis communication and behavior [31]. Collectively, these results suggest that the antidepressant effect of SNS is linked to the regulation of the tryptophan metabolism pathway in the prefrontal cortex.

### SNS significantly enriches IAA levels in colon and prefrontal cortex tissue

**Fig. 3:**
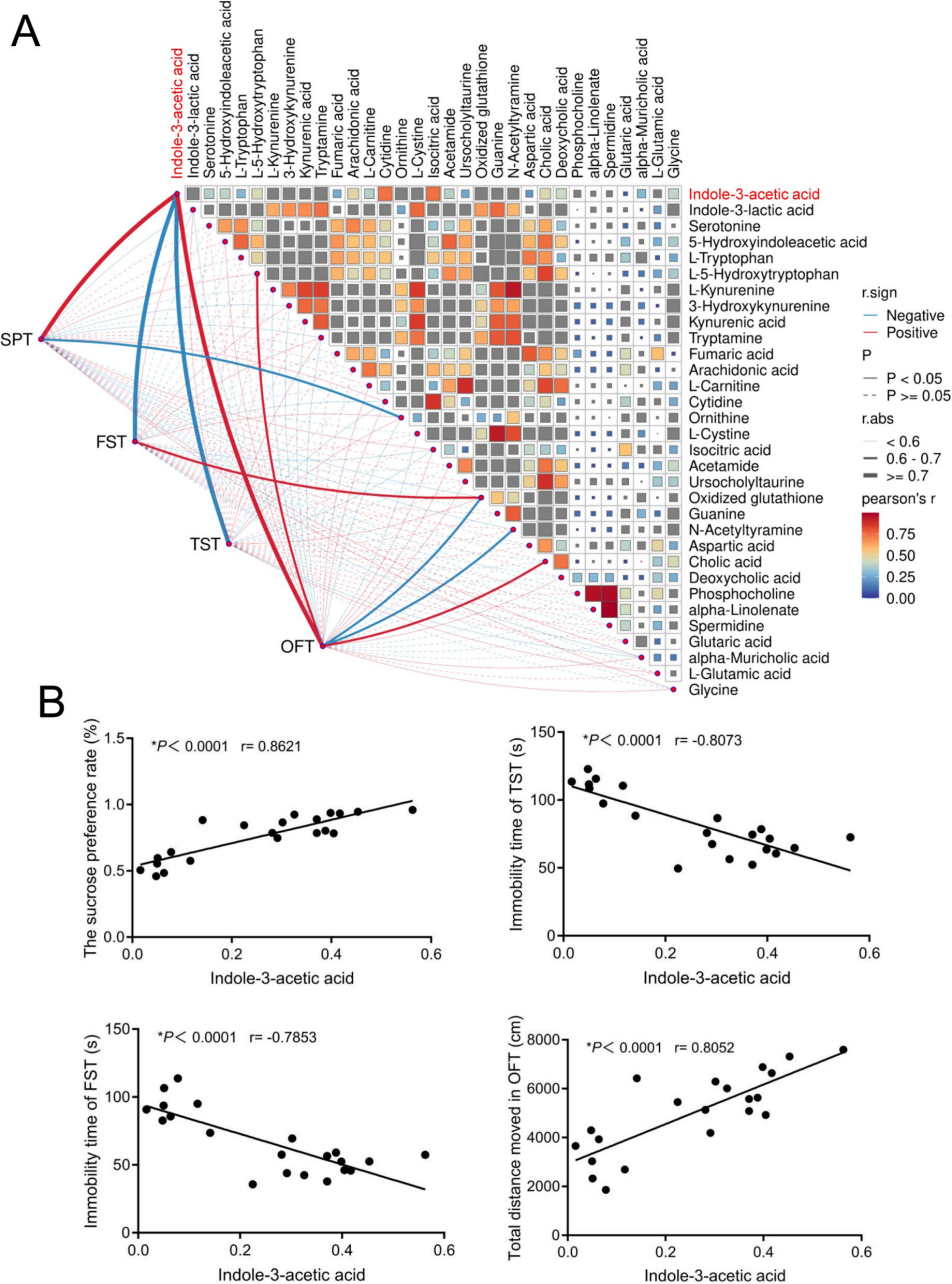
The correlation analysis between the differential metabolites and depression-related behavioral indicators. **A** The correlation analysis between the differential metabolites and depression-related behavioral indicators was performed using the OmicStudio_Cor_link tool. **B** Scatter plot depicting the relationship between prefrontal cortex IAA levels and depression-related behavioral indicators. Pearson/Spearman correlation coefficients were used to evaluate the associations

To identify key metabolites associated with depression, we performed Pearson/Spearman correlation analysis between the differential metabolites and depression-related behavioral indicators. The correlation analysis revealed that IAA levels in the prefrontal cortex showed significant positive correlations with sucrose preference rate (r = 0.8621, P < 0.0001) and total distance moved in the OFT (r = 0.8052, *P* < 0.0001). Conversely, IAA levels showed a significant negative correlation with the immobility time in both the FST (r = −0.7853, *P* < 0.0001) and the TST (r = −0.8073, *P* < 0.0001) (Fig. 3A, B). These results suggest that IAA may be a critical signaling molecule mediating the antidepressant effects of SNS.

**Fig. 4:**
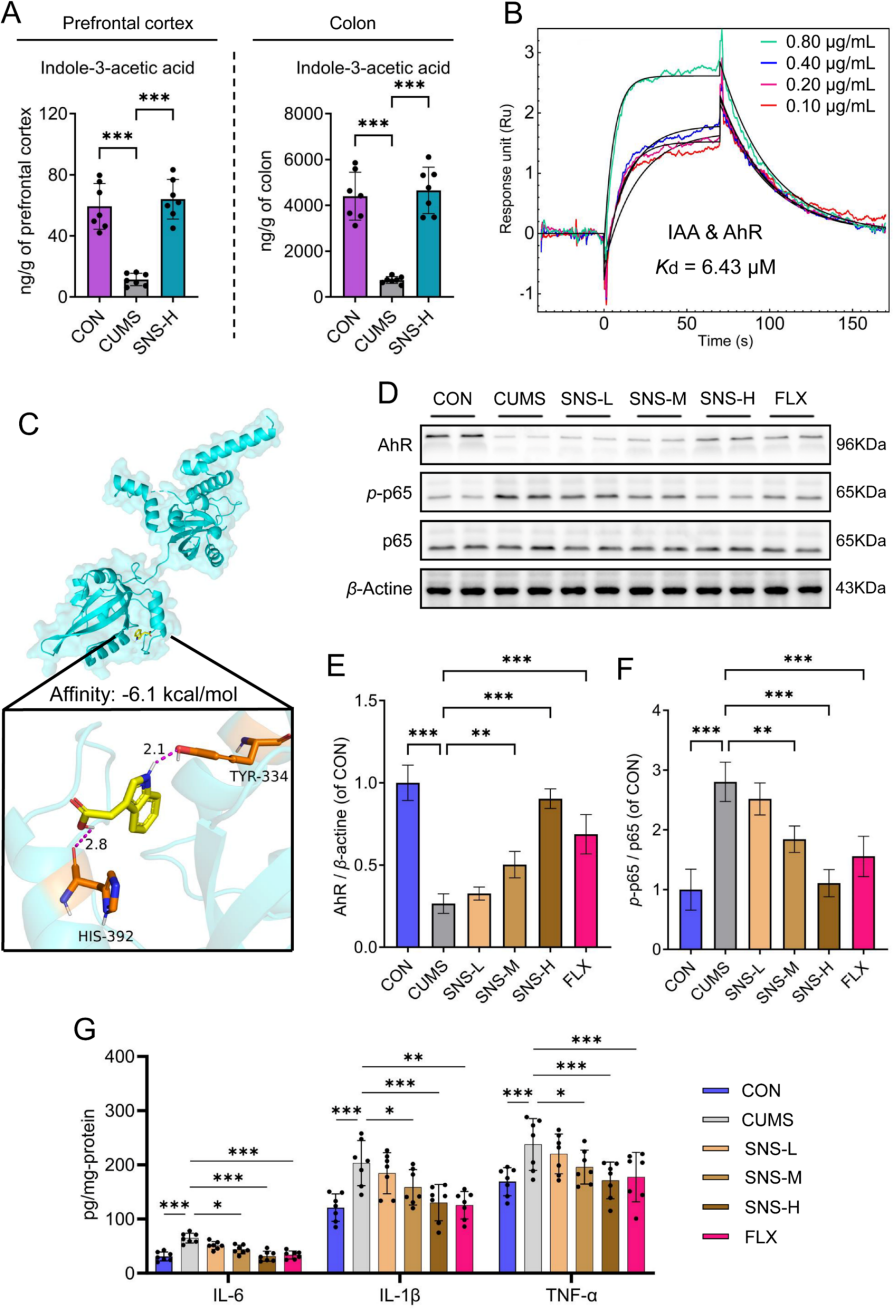
SNS modulates gut microbiota-driven IAA to alleviate depression via AhR/NF-κB signaling. **A** The levels of IAA in both the prefrontal cortex and colon were detected using UHPLC-Q-TRAP 6500/MS (*n* = 7). **B** Direct affinity determination of IAA for the AhR via surface plasmon resonance (SPR) analysis. **C** Molecular docking analysis of the predicted binding mode between IAA and the AhR. **D** Western blot analysis of AhR, *p*-p65, and p65 protein levels in prefrontal cortex tissues from the CON, CUMS, SNS-L, SNS-M, SNS-H, and FLX groups (*n* = 4). **E** Quantitative densitometric analysis of AhR protein levels in prefrontal cortex tissues from the CON, CUMS, SNS-L, SNS-M, SNS-H, and FLX groups (*n* = 4). **F** Quantitative densitometric analysis of the ratio of *p*-p65 to p65 in prefrontal cortex tissues (*n* = 4). **G** Protein levels of pro-inflammatory cytokines IL-1*β*, IL-6, and TNF-*α* in the prefrontal cortex were measured by ELISA (*n* = 7). All results are shown as mean ± SD. *P* values were computed via two-tailed Student’s t-test (between two groups) or one-way ANOVA (among several groups). ^*^P < 0.05, ^**^P < 0.01, and ^***^P < 0.001, vs CUMS group

Given that IAA is a product of microbial tryptophan metabolism, produced by the gut microbiota and capable of crossing the blood–brain barrier [32], we next sought to investigate the interaction between SNS and the gut microbiome. To determine whether the antidepressant effects of SNS are dependent on the gut microbiota, we performed a gut microbiota depletion experiment using a broad-spectrum antibiotic cocktail. Behavioral assessments revealed that while SNS treatment significantly ameliorated CUMS-induced depressive-like behaviors, as evidenced by increased sucrose preference in the SPT, reduced immobility time in the FST and TST, and increased total distance moved in the OFT, these antidepressant effects were largely abolished in mice receiving concurrent antibiotic treatment (SNS + ABX group; Supplementary Fig. S4). This indicates that gut microbiota depletion attenuates the antidepressant efficacy of SNS. Subsequently, using a previously validated targeted metabolomics approach [30], we quantified IAA levels in both the prefrontal cortex and colon. The results demonstrated that the levels of microbiota-derived IAA were significantly reduced in both the prefrontal cortex and colon of CUMS mice. Notably, SNS treatment markedly increased IAA levels in both colon and prefrontal cortex tissues (Fig. 4A). This suggests that the regulation of IAA levels may be a key mechanism through which SNS exerts its antidepressant effect.

### SNS exerts antidepressant effects via the gut microbiome-derived IAA-AhR/NF-κB pathway

Previous studies have established that gut microbiota-derived indole derivatives, including IAA, can activate the AhR in the gut to exert diverse biological effects, such as alleviating DSS-induced colitis, modulating intestinal stem cell renewal and tumorigenesis, and mitigating intestinal aging [21, 24, 33, 34] Furthermore, activation of AhR in the brain has been reported to confer antidepressant effects by suppressing NF-κB pathway-mediated neuroinflammation [10, 11, 30, 35, 36]. Critically, gut microbiome-derived IAA has been demonstrated to cross the blood–brain barrier [37]. Based on these findings, we hypothesize that SNS alleviates depression through enriching gut microbiota-derived IAA, which serves as a potential ligand for brain AhR and, after crossing the blood–brain barrier, activates prefrontal cortical AhR to suppress NF-κB-mediated neuroinflammation.

Guided by this evidence, we analyzed the IAA-AhR/NF-κB pathway to determine whether the antidepressant efficacy of SNS is mediated by gut microbiome-derived IAA through activation of AhR in the prefrontal cortex. Surface plasmon resonance (SPR) analysis demonstrated a binding affinity between IAA and AhR with a dissociation constant (*K*_d_) of 6.43 μM (Fig. 4B), which was further corroborated by molecular docking showing that IAA binds directly to AhR via residues such as Tyr334 and His392, with a binding free energy of -6.1 kcal/mol (Fig. 4C and Supplementary Fig. S5). Based on these findings, we assessed the protein expression levels of AhR and the NF-κB pathway components in the prefrontal cortex. Western blot analysis revealed that, compared to the CON group, the CUMS group exhibited significantly downregulated AhR expression and an increased *p*-p65/p65 ratio, both of which were dose-dependently reversed by SNS treatment (Fig. 4D–F), indicating that SNS inhibits CUMS-induced NF-κB activation via IAA-mediated AhR signaling. Moreover, ELISA results showed that SNS significantly suppressed the production of pro-inflammatory cytokines, including IL-1β, IL-6, and TNF-α, in the prefrontal cortex of CUMS mice (Fig. 4G). These results suggest that SNS alleviates CUMS-induced neuroinflammation by enriching gut microbiota-derived IAA, which activates AhR and subsequently inhibits the NF-κB pathway in the prefrontal cortex. In summary, these findings highlight the unique role of gut microbiome-derived IAA as a gut–brain signaling mediator underlying the antidepressant mechanism of SNS.

### SNS ameliorates gut microbiota dysbiosis and increases intestinal IAA production by enriching *Lactobacillus*

**Fig. 5:**
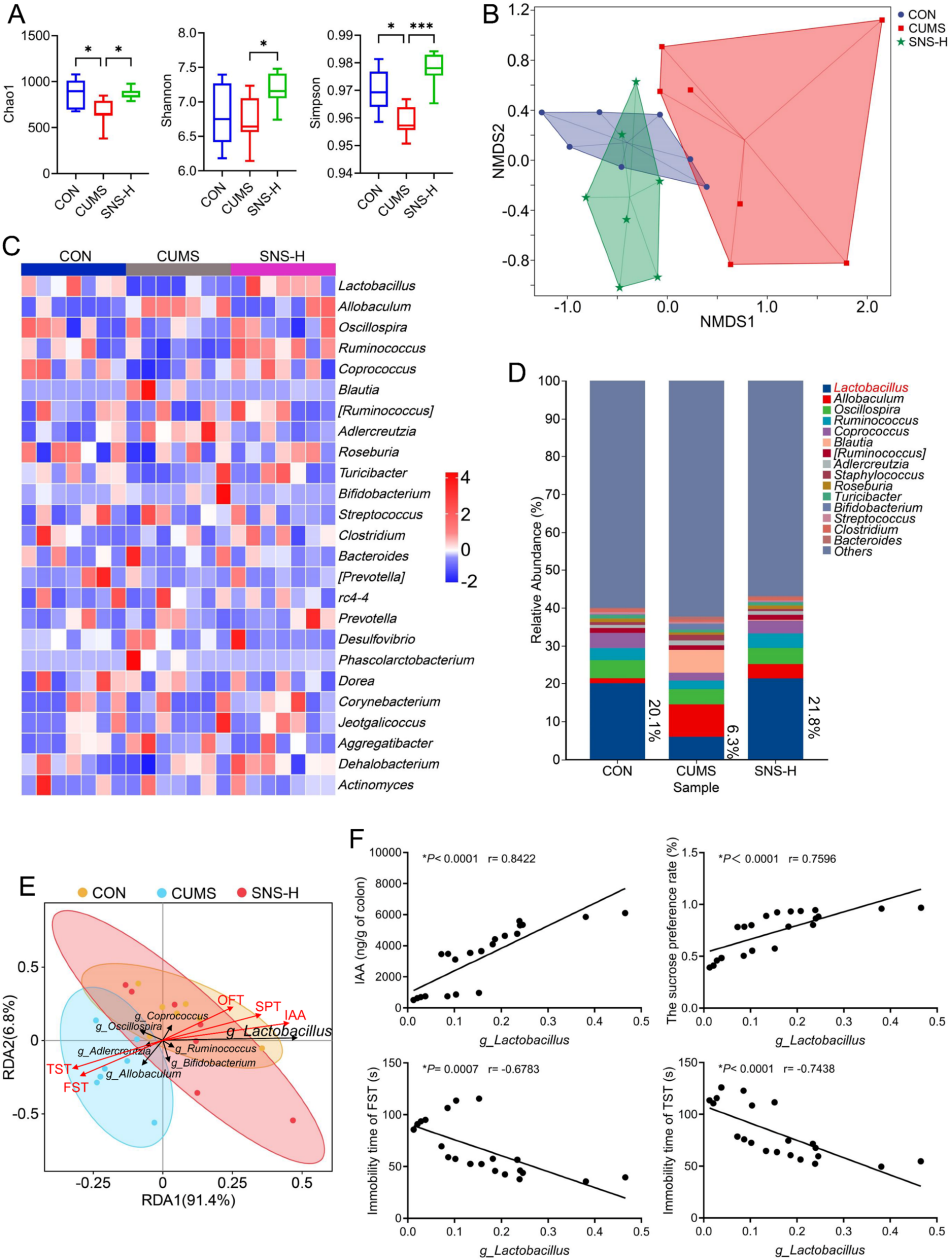
SNS increased IAA production potentially through enriching *Lactobacillus*. **A** Alpha diversity indices (Chao1, Shannon, Simpson) of the gut microbiota from the CON, CUMS, and SNS-H groups. **B** Non-metric multidimensional scaling (NMDS) plot based on Bray–Curtis dissimilarities to visualize beta-diversity among the CON, CUMS, and SNS-H groups. **C** Heatmap of gut microbiota at the genus level responsive to SNS treatment. **D** Taxonomic composition of the gut microbiota analyzed at the genus level. **E** Redundancy analysis (RDA) assessing the relationship between significantly altered gut microbiota and depression-associated symptoms (SPT, FST, TST, OFT, and colon IAA levels). **F** Scatter plot illustrating correlations derived from Pearson/Spearman coefficients. All results are shown as mean ± SD (*n* = 7). *P* values were computed via two-tailed Student’s t-test (between two groups) or one-way ANOVA (among several groups). ^*^P < 0.05, ^**^P < 0.01, and ^***^P < 0.001, vs CUMS group

Previous studies have identified IAA as a microbial metabolite derived from tryptophan metabolism, primarily produced by IAA-generating gut bacteria such as *Lactobacillus* and *Clostridium* species [31]. However, the specific mechanism through which SNS modulates IAA-producing bacteria to elevate IAA levels in the colon and prefrontal cortex remained unclear. To address this, we performed 16S rRNA sequencing to assess the regulatory effects of SNS on the gut microbiota. The results revealed that alpha diversity indices (including the Chao and Simpson indices) were significantly reduced in the CUMS-induced depression model group compared to the CON group, while SNS-H treatment markedly restored gut microbial alpha diversity (Fig. 5A). Non-metric multidimensional scaling (NMDS) analysis indicated that SNS-H shifted the gut microbial composition from the CUMS-associated profile toward that of the CON group (Fig. 5B). Furthermore, notable differences in gut microbiome composition and abundance were observed among the CON, CUMS, and SNS-H groups (Fig. 5C and Supplementary Fig. S6). Subsequently, LEfSe analysis based on a Linear Discriminant Analysis (LDA) score threshold > 2 was conducted to identify differentially abundant bacterial taxa between the CON and CUMS groups, as well as between the SNS-H and CUMS groups. This analysis revealed 27 and 34 differentially abundant bacterial taxa in the CON vs. CUMS and SNS-H vs. CUMS comparisons, respectively (Supplementary Fig. S7). Notably, at the genus level, the relative abundance of *Lactobacillus* was significantly reduced in CUMS-induced depressed mice, declining from 20.1% in the CON group to 6.3% in the CUMS group. This reduction was effectively reversed by SNS-H treatment, which restored the relative abundance of *Lactobacillus* to 21.8% (Fig. 5D). Among all observed taxa, *Lactobacillus* exhibited the most pronounced alterations across groups. In addition, redundancy analysis (RDA) and Pearson/Spearman correlation analyses confirmed that *Lactobacillus* abundance was positively correlated with colon IAA levels (r = 0.8422, *P* < 0.0001), sucrose preference rate (r = 0.7596, *P* < 0.0001), and total distance moved in the OFT (r = 0.6220, *P* = 0.0026), and negatively correlated with immobility time in both the FST (r = −0.6783, *P* = 0.0007) and TST (r = −0.7438, *P* < 0.0001) (Fig. 5E, F and Supplementary Fig. S8). It is noteworthy that *Lactobacillus* has been reported to exhibit tryptophan 2-monooxygenase and indoleacetamide hydrolase activities, enabling the conversion of tryptophan to IAA in the gut [12]. Taken together, these findings demonstrate that SNS enhances intestinal IAA production by ameliorating gut microbiota dysbiosis and specifically enriching *Lactobacillus*.

### Gut microbiome-derived IAA supplementation alleviates depression-like behavior via the AhR/NF-κB pathway

**Fig. 6:**
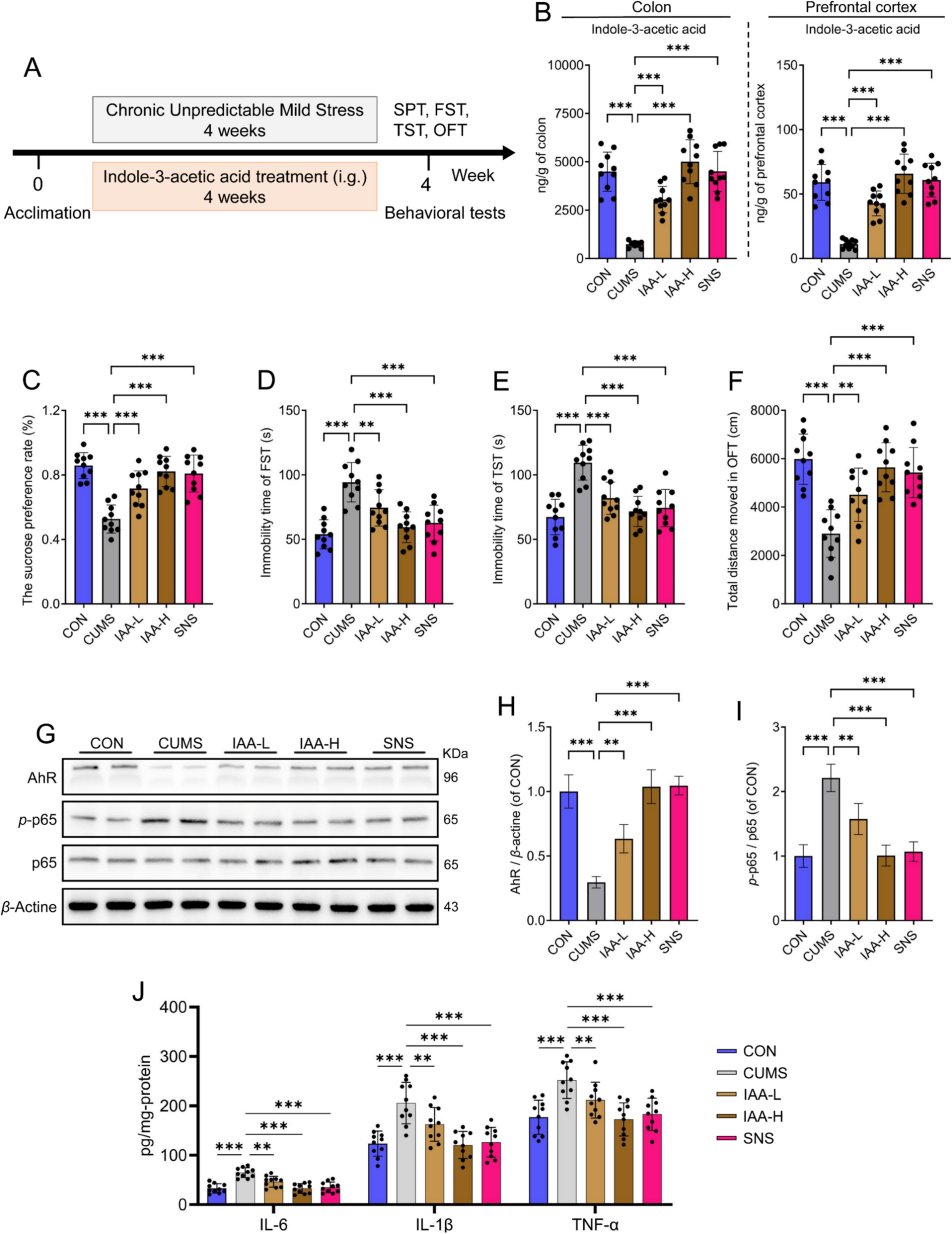
Gut microbiome-derived IAA regulates depression-like behavior via the AhR/NF-κB pathway. **A** Experimental timeline for the IAA supplementation study (*n* = 10). **B** The levels of IAA in both the prefrontal cortex and colon were detected using UHPLC-Q-TRAP 6500/MS. **C** Sucrose preference of the IAA supplementation study. **D** Immobility time of mice in the forced swim test (FST). **E** Immobility time of mice in the tail suspension test (TST). **F** Total distance moved in the open field test (OFT). **G** Western blot analysis of AhR, *p*-p65, and p65 protein levels in prefrontal cortex tissues from the CON, CUMS, IAA-L, IAA-H, and SNS groups (*n* = 4). **H** Quantitative densitometric analysis of AhR protein levels in prefrontal cortex tissues from the CON, CUMS, IAA-L, IAA-H, and SNS groups (*n* = 4). **I** Quantitative densitometric analysis of the ratio of *p*-p65 to p65 in prefrontal cortex tissues (*n* = 4). **J** Protein levels of pro-inflammatory cytokines IL-1*β*, IL-6, and TNF-*α* in the prefrontal cortex were measured by ELISA (*n* = 10). All results are shown as mean ± SD. *P* values were computed via two-tailed Student’s t-test (between two groups) or one-way ANOVA (among several groups). ^**^P < 0.01, and ^***^P < 0.001, vs CUMS group

To investigate whether IAA serves as a gut-brain signaling messenger mediating the antidepressant effects of SNS, CUMS mice were orally administered IAA for 4 weeks, and its antidepressant efficacy, along with its impact on the AhR/NF-κB pathway, was assessed (Fig. 6A). In this experiment, SNS was used as a positive control. As expected, oral administration of IAA significantly increased IAA levels in both colonic content and prefrontal cortical tissue (Fig. 6B), supporting the possibility that gut microbiota-derived IAA can enter the systemic circulation and subsequently influence depression-like behavior by reaching the brain. Consistent with the effects of SNS, IAA treatment markedly increased the sucrose preference rate and total distance moved in the OFT, while significantly reducing immobility time in both the FST and TST in CUMS mice (Fig. 6C–F). Collectively, these data demonstrate that IAA exerts antidepressant-like effects comparable to those of SNS.

**Fig. 7:**
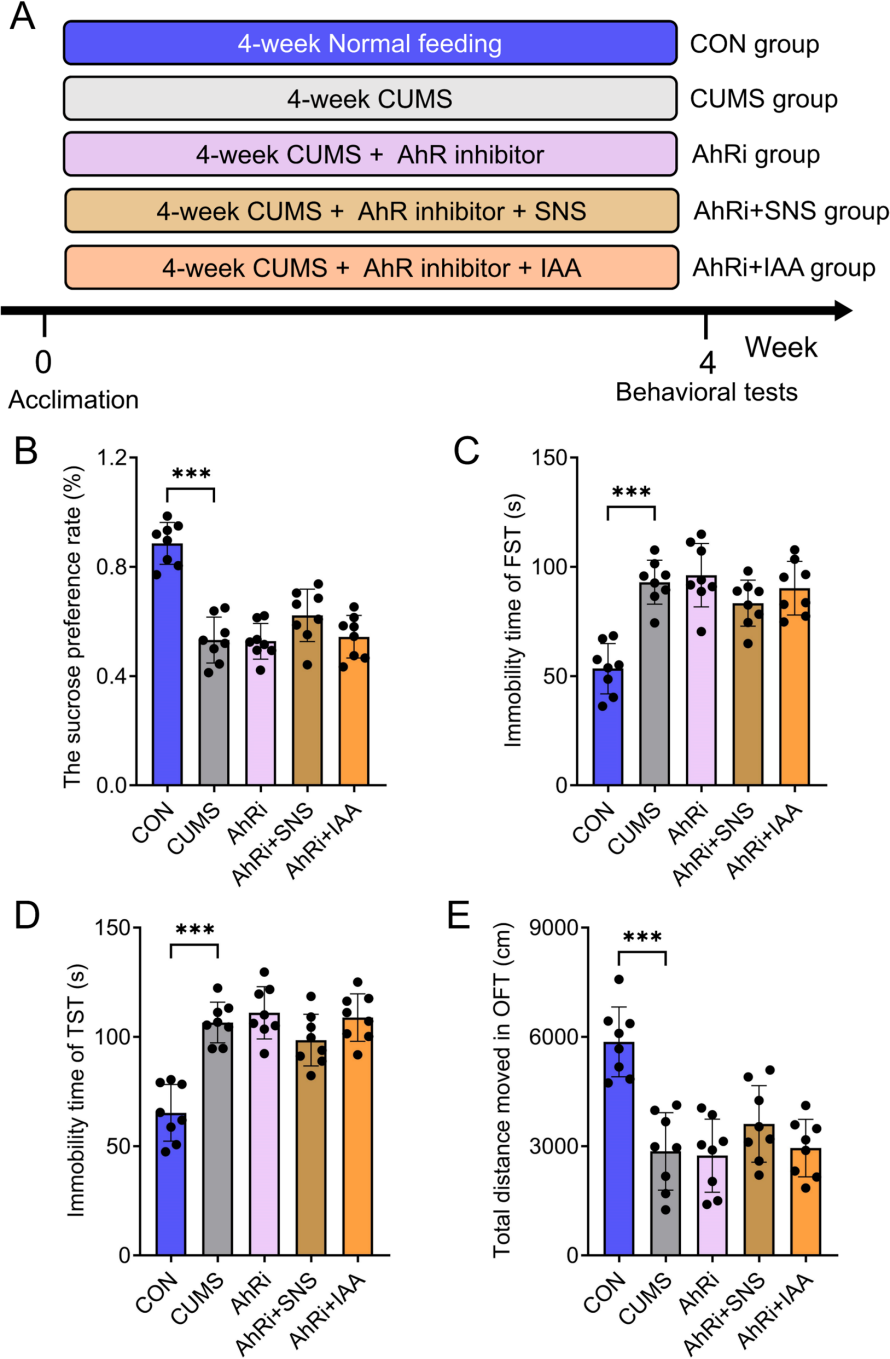
AhR antagonist rescue experiment validates the essential role of AhR in mediating the antidepressant effects of SNS and IAA. **A** Timeline of experimental procedures. **B** Sucrose preference rate in the SPT. **C** Immobility time recorded in the FST. **D** Immobility time recorded in the TST. **E** Total movement distance in the OFT. All results are shown as mean ± SD (*n* = 8). *P* values were computed via two-tailed Student’s t-test (between two groups) or one-way ANOVA (among several groups). ^***^P < 0.001, vs CUMS group

To further validate that both SNS and its gut-brain signal, IAA, alleviate depression via the AhR/NF-κB pathway, we measured protein levels of AhR, *p*-p65, and p65 in the prefrontal cortex following treatment with IAA or SNS. In line with the results observed with SNS, IAA significantly upregulated AhR expression and reduced the *p*-p65/p65 ratio (Fig. 6G–I). Moreover, IAA pretreatment substantially decreased the levels of pro-inflammatory cytokines IL-1*β*, IL-6, and TNF-*α* in the prefrontal cortex, thereby mitigating CUMS-induced neuroinflammation (Fig. 6J). To further establish the causal role of AhR in mediating the antidepressant effects of SNS and IAA, a pharmacological rescue experiment was conducted using the specific AhR inhibitor Stemregenin 1. Consistently, AhR inhibition completely abolished the antidepressant effects of both SNS and IAA, as evidenced by the reversal of CUMS-induced behavioral improvements in sucrose preference rate, immobility time in the FST and TST, and total distance moved in the OFT (Fig. 7A–E). These results functionally confirm that the AhR/NF-κB-mediated neuroinflammatory pathway is involved in IAA's antidepressant mechanism.

## Discussion

MDD is a highly prevalent psychiatric condition whose pathophysiology has not been fully elucidated [38, 39]. Emerging evidence highlights the gut-brain axis as a critical interface in the development and progression of depression [40]. Nevertheless, the precise pathways through which intestinal microbiota affect brain function and shape antidepressant treatment outcomes are still not well understood. This study aimed to elucidate the mechanism by which SNS, a classic Traditional Chinese Medicine formula, alleviates depression-like behavior. Our findings demonstrate, for the first time, that the antidepressant effects of SNS are not mediated by direct central action but rather by remodeling the gut microbiota, leading to increased production of the microbial tryptophan metabolite IAA, which subsequently suppresses neuroinflammation in the prefrontal cortex via the AhR/NF-κB pathway. Likewise, our findings underscore the crucial role of the gut microbiota within this IAA-AhR/NF-κB axis in ameliorating depression-like behaviors.

To characterize the specific changes in the gut microbiota following SNS administration, we conducted 16S rRNA sequencing and observed that CUMS exposure significantly reduced gut microbial alpha diversity and altered community structure, which were effectively reversed by high-dose SNS treatment. Notably, SNS specifically enriched the genus Lactobacillus, whose relative abundance was markedly decreased in CUMS mice and robustly restored by SNS-H. This shift is particularly important, as numerous studies have identified Lactobacillus as a key producer of IAA via tryptophan metabolism, possessing tryptophan 2-monooxygenase and indoleacetamide hydrolase activities [12]. The positive correlation between Lactobacillus abundance and colonic IAA levels, coupled with the observed increase in IAA in both the colon and PFC after SNS treatment, strongly suggests that SNS enhances IAA production by selectively promoting the growth of bacteria that synthesize IAA. Despite these findings, it remains unclear which specific components of the multi-herb formula SNS are primarily responsible for modulating Lactobacillus. Given the complexity of herbal formulations, further studies are warranted to identify the active constituents driving this effect, which would be crucial for the development of microbiota-targeted antidepressant therapies. Elucidating the mechanisms of action of Traditional Chinese Medicine (TCM) formulas and the modern characterization of TCM theory are complex issues. Growing evidence suggests that the gut microbiota may serve as a significant biological basis for their effects, and our findings bridge TCM and modern microbiology. Furthermore, the mechanism by which IAA functions as a key gut-brain messenger remained unresolved.

IAA is an important indole-derived metabolite [41] and a potential gut-brain messenger produced by the microbiota that influences host physiology 4 [34]. Substantial evidence indicates that indole derivatives of tryptophan metabolism, including IAA, are important specific ligands for AhR [42]. AhR is a ligand-activated transcription factor that plays a central role in immunoregulation, cell differentiation, and xenobiotic metabolism. It can exert antidepressant effects through various pathways, such as inhibiting canonical pro-inflammatory signaling pathways, like NF-κB, and ameliorating neuroinflammation [36]. In our study, we detected coordinated increases in IAA levels in both the colon and PFC following SNS administration, consistent with previous reports that gut microbiota-derived IAA can cross the blood–brain barrier [32]. To establish a direct link between IAA and its central molecular target, we employed surface plasmon resonance (SPR) and molecular docking. These experiments confirmed that IAA binds directly to AhR with appreciable affinity, forming specific interactions with residues such as Tyr334 and His392. This binding event is functionally significant, as activation of AhR in the brain has been shown to suppress NF-κB-driven neuroinflammation, a well-established contributor to depression [43]. Accordingly, we found that SNS treatment dose-dependently upregulated AhR expression and reduced NF-κB p65 phosphorylation in the prefrontal cortex, concomitant with decreased levels of the pro-inflammatory cytokines IL-1*β*, IL-6, and TNF-*α*. This study provides novel insights into the therapeutic role of gut microbiota and their metabolites in regulating depression-like behavior.

While our metabolomic analysis revealed that SNS treatment also modulated other tryptophan metabolites—increasing HIAA and decreasing KYNA and ILA—these changes should be interpreted within the context of their physiological relevance. Correlation analysis demonstrated that among all detected metabolites, only IAA levels showed strong and consistent correlations with multiple depression-related behavioral parameters (Fig. 3), suggesting that IAA is the key metabolite functionally linked to the antidepressant effects of SNS. Regarding HIAA and KYNA, although both can potentially activate AhR signaling, previous studies have shown that they function as AhR ligands only at supraphysiological concentrations, whereas IAA is a physiologically relevant endogenous AhR agonist that binds competitively to AhR at concentrations achievable in the brain [12, 44, 45]. The observed decrease in ILA following SNS-H treatment is particularly noteworthy, as recent findings demonstrate that increased ILA coupled with decreased indole-3-carboxaldehyde (IAld) promotes depressive-like behavior, while reduced ILA alleviates depressive symptoms [46]. Thus, the reduction in ILA observed in our study aligns with previous findings that reduced ILA alleviates depressive symptoms. Collectively, these findings indicate that although SNS modulates multiple nodes of tryptophan metabolism, the specific pattern of changes—particularly the robust increase in IAA and decrease in ILA—converges to support the amelioration of depressive-like behaviors through coordinated effects on AhR signaling and neuroplasticity.

This study provides a novel mechanism for the antidepressant effects of SNS at the gut-brain axis. Specifically, SNS alleviates neuroinflammation and exerts antidepressant efficacy by specifically enriching intestinal Lactobacillus, thereby modulating the production of the tryptophan metabolite IAA, which ultimately acts through the AhR/NF-κB signaling pathway. It is important to note that tryptophan is also metabolized via the kynurenine pathway and serves as the precursor for serotonin synthesis, both of which are implicated in mood regulation [9, 31]. However, in the context of CUMS-induced depression, our multi-omics and correlation analyses consistently pointed to the IAA-AhR axis as the predominant pathway modulated by SNS. This may be because microbial-derived IAA directly engages the central AhR to suppress neuroinflammation, a core pathological process in depression [11], whereas serotonin synthesis in the brain is more tightly regulated by host enzymatic activity and may not be as directly influenced by gut microbial changes [9]. Nevertheless, the potential interplay between these pathways warrants further investigation. This work not only provides a detailed mechanistic explanation for the efficacy of a TCM formula via the microbiota-gut-brain axis but also highlights IAA and IAA-producing probiotics (e.g., *Lactobacillus*) as potential therapeutic targets or agents for depression treatment. However, certain limitations must be acknowledged. Although our correlative and interventional data are compelling, further experimental evidence is required to definitively establish causality. Moreover, the genus *Lactobacillus* comprises numerous species, and the specific species responsible for IAA production in this context warrants further identification. Future studies should also aim to identify the active components within SNS that drive Lactobacillus enrichment and validate this pathway in clinical populations.

## Conclusion

In conclusion, our study demonstrates that the antidepressant effect of SNS is mediated through a gut-initiated signaling cascade involving the enrichment of *Lactobacillus*, elevation of microbial IAA levels, activation of central AhR, and subsequent inhibition of NF-κB-mediated neuroinflammation. Crucially, these findings establish that gut microbiome-derived IAA plays a pivotal role as a key gut-brain signal in this process. Collectively, these findings not only elucidate a novel mechanism of SNS but also underscore the potential of targeting the gut microbiome and its metabolites as a strategic avenue for developing novel antidepressant therapies.

## Supplementary Information

Supplementary material 1.

## Data Availability

The data used in this study can be provided in accordance with requests.

## Abbreviations

AhR: Aryl hydrocarbon receptor
CE: Collision energy
CUMS: Chronic unpredictable mild stress
DP: Declustering potential
FST: Forced swim test
IAA: Indole-3-acetic acid
OFT: Open field test
OPLS-DA: Orthogonal partial least squares discriminant analysis
PCA: Principal component analysis
PCoA: Principal coordinates analysis
SNS: Si-Ni-San
SPR: Surface plasmon resonance
SPT: Sucrose preference test
TST: Tail suspension test
