# Supplementary material for: Si-Ni-San alleviates depression-like behavior via regulating the gut microbiota-tryptophan metabolism-AhR/NF-κB pathway axis
Source: Chin Med. 2026 Apr 7;21:111. doi: 10.1186/s13020-026-01390-4 (PMC13054972; doi:10.1186/s13020-026-01390-4)
Supplement: Supplementary file 1 — Supplementary material 1. [file 13020_2026_1390_MOESM1_ESM.docx]

**Si-Ni-San alleviates depression-like behavior via regulating the gut microbiota-****tryptophan metabolism****-AhR/NF-κB pathway axis**

Qiang Xiao ^a, b^^†^, Zhaoyi Wen ^a, b†^, Huang Zhan ^a, b^, Han Zhao ^a, b^, Yukun Jiao ^a, b^, Dehua Huang ^c, *^, Hui Li ^a, b, d, *^, Congcong Chen ^a, b^^,^ ^*^

^a^ *Jiangxi Province Key Laboratory of Traditional Chinese Medicine Pharmacology, Institute of Traditional Chinese Medicine Health Industry, China Academy of Chinese Medical Sciences,* *Nanchang 330115, China;*

^b^ *Jiangxi Health Industry Institute of Traditional Chinese Medicine, Nanchang 330115, China;*

^c^ *Modern Research Center for Traditional Chinese Medicine, The Key Laboratory of Chemical Biology and Molecular Engineering of Ministry of Education, Shanxi University, Taiyuan, 030006, China.*

^d^ *Institute of Chinese Materia Medica, China Academy of Chinese Medical Sciences, Beijing 100700, China.*

**^*^** Corresponding authors.

*E-mail addresses*: [dehuahedu@126.com](mailto:dehuahedu@126.com) (D. Huang); [lihuiyiren@163.com](mailto:lihuiyiren@163.com) (H. Li) [chencongcong@itcmhi.ac.cn](mailto:chencongcong@itcmhi.ac.cn) (C. Chen)

^†^ These authors have contributed equally to this work.

**Supplemental File:**

**Supplemental File 1. Chromatographic analysis of SNS by HPLC**

*1.1 Sample preparation*

The four component herbs—Radix Bupleuri, Radix Paeoniae Alba, Fructus Aurantii Immaturus, and Radix et Rhizoma Glycyrrhizae—were weighed in equal proportions (1:1:1:1) and immersed in distilled water (1:10, *w/v*) for 2 hours. The mixture was subsequently reflux-extracted twice using the same volume of water, each extraction lasting 2 hours. The resulting solutions were combined, concentrated under reduced pressure, and lyophilized to yield a powdered extract, with a final yield of 11.12%.

An accurately weighed sample of 1.0 g dried powdered sample of SNS was introduced with methanol in a 10 ml volumetric flask. A volume of 2 ml of the solution was filtered through a 0.22-mm syringe filter before use. A 1-ml volume of the solution was injected for HPLC analysis.

*1.2 Chromatography analysis*

An ACQUITYTM H-Class UPLC system (Waters, Milford, MA, USA) equipped with an ACQUITY HSS T3 column (2.1×100 mm,1.8 μm) maintained at 35°C was applied for detection of SNS. The mobile phase consisted of 0.1% FA-water (A) and 0.1% FA-acetonitrile (B) running at a flow rate of 0.3 mL/min. The injection volume was 2 μL and the PDA detector was set at 260 nm. The optimized elution gradient program was set as follows: 0–0.5 min, 5% (B); 0.5-2 min, 5-20% (B); 2-8 min, 20-21% (B); 8-12 min, 21-22% (B); 12-16 min, 22-25% (B); 16-18 min, 25-60% (B) ; 18-20 min, 60-100% (B); 20-21 min, 100% (B); 21-21.5 min, 100-5% (B); 21.5-25 min, 5% (B).

Taking paeoniflorin, liquiritin, naringin, hesperidin as chemical marker, the identified results are shown in Fig. S1.

**Supplemental File 2. Behavior test of sucrose preference test (SPT), tail suspension test (TST), forced swim test (FST), and open-field test (OFT)**

*2.1 Sucrose preference test (SPT)*

Before the experiment, each mice was given 1% sucrose solution and daily drinking water for the training of the sucrose preference test. Firstly, two bottles of 1% sucrose solution were given for the first 24 hours. Secondly, one bottle of 1% sucrose solution and other daily drinking water were given for the second 24 hours to train all mice. After the end of the training, the water and food were deprived for 12 h, and then the SPT experimented within 12 hours, the mice were free to choose 1% sucrose solution or water during the test. The weight of the consumed sucrose solution and the water was measured to calculate the sucrose preference rate of the mice. The sucrose preference rate = the weight of consumed sucrose solution/ (the weight of consumed sucrose solution + the weight of consumed water).

*2.2 Forced swim test (FST)*

The forced swimming test (FST) was conducted to evaluate behavioral despair in mice, wherein the duration of immobility after initial escape-oriented movements is considered to reflect depression-like behavior. Briefly, each mouse was placed individually in a transparent glass or plastic cylinder (25–30 cm in height, 15–20 cm in diameter) filled with water to a depth of approximately 15 cm, maintained at 23–25°C. The test consisted of a 15-minute pre-swim session, after which the mouse was gently removed, dried with a towel, and placed under a heating lamp or dried with a hairdryer to prevent hypothermia before being returned to its home cage. Twenty-four hours later, the mouse was subjected to a 7-minute test session in the same apparatus. The final 5 minutes of the test session were recorded, with the first 2 minutes considered an acclimation period and excluded from analysis. The total immobility time during the remaining 4 minutes was analyzed using the SMART 3.0 behavioral tracking software. Immobility was defined as the point when the mouse ceased struggling and remained floating vertically in the water, making only minimal movements necessary to keep its head above the surface.

*2.3 Tail Suspension Test (TST)*

The tail suspension test (TST) was employed to assess behavioral despair by measuring the duration of immobility when mice were suspended by their tails. Each mouse was affixed with adhesive tape approximately 1–2 cm from the tip of the tail to a horizontal hook on the top panel of the test box, such that the mouse was suspended upside down with its head positioned 10–15 cm above the floor. The test session lasted 7 minutes and was recorded in its entirety. The total immobility time during the last 5 minutes was quantified using the SMART 3.0 video tracking system. Immobility was defined as the absence of escape-oriented movements, with the mouse hanging passively and motionless, except for minor involuntary limb movements.

*2.4 Open-field test (OFT)*

The open field test (OFT) was performed to evaluate locomotor activity and anxiety-like behavior in mice, as decreased exploration of the central zone is indicative of heightened anxiety levels. The apparatus consisted of a square arena (40 cm × 40 cm × 40 cm) with its floor virtually divided into a central and a peripheral area. A video camera mounted above the arena was connected to a computer for behavioral recording. At the beginning of each trial, a mouse was gently placed in the same corner of the arena (e.g., the southwest corner) facing the wall and allowed to explore freely for 5 minutes. The session was recorded, and parameters including total distance traveled, time and distance spent in the central zone, average speed, and number of rearings were analyzed using SMART 3.0 software. After testing, each mouse was returned to its home cage, and the arena was thoroughly cleaned to eliminate olfactory cues. Total distance moved and average speed were used to assess general locomotor activity, while time and distance in the center zone served as indicators of anxiety-like behavior, with less center activity reflecting higher anxiety.

**Supplemental File 3.** **Collection and** **preparation of** **prefrontal cortex tissues and** **colon contents** **samples for UHPLC-MS/MS analysis**

*3.1 Collection and preparation of* *prefrontal cortex tissues*

Collection of prefrontal cortex tissue samples: The mouse were sacrificed 1 h after the last intragastric administration. Samples of prefrontal cortex tissue samples were collected immediately after the sacrifice. The prefrontal cortex tissues were freeze-dried, ground into powder on ice, and mixed homogeneously for further analysis. All extraction solvents were kept at 4 °C before adding to the samples. The sample/solvent value was 1/5 (mg of prefrontal cortex tissues /μL of solution). After that, 20 mg of prefrontal cortex tissue powder was homogenized for 1 min in chilled methanol/water (1:1, *v/v,* 100 μL), vortexed for 2 min, and centrifuged at 4 °C and 13,000 rpm for 15 min. The supernatant was transferred to a Eppendorf tube, and the precipitate was re-extracted in the same manner, combining the two extracted supernatants together to a final volume of 200 μL. Next, the supernatant was dried under N_2_ before analysis.

Preparation of prefrontal cortex tissue samples: The dry extract was reconstituted in 100 μL methanol-water (4:1, *v/v*) containing the two IS (1 μg/mL of each IS, yohimbine and reserpine), vortexed for 2 min, and centrifuged at 4 °C/13,000 rpm, for 10 min. Finally, 80 μL supernatant was transferred to autosampler vials as a test sample. Besides, 8 μL from each test sample were thoroughly mixed to create a pooled prefrontal cortex sample, used as a quality control (QC) sample. For the QC sample, the analytical method was the same as the test samples, and the QC sample was inserted for every ten test samples to check the stability and performance of the instrument.

*3.2 Collection and preparation of colon contents samples*

Collection of colonic contents samples: The mouse were sacrificed 1 h the last intragastric administration. Samples of colonic contents (from the middle segment of the colon) were collected immediately after sacrifice. The colonic contents were freeze-dried, ground into powder on ice, and mixed homogeneously for further analysis. All extraction solvents were kept at 4 °C before adding to the samples. The sample/solvent value was 1/10 (mg of colonic contents/μL of solution). After that, 20 mg of colonic contents powder was homogenized for 1 min in chilled methanol/water (1:1, *v/v*, 200 μL), vortexed for 2 min, and centrifuged at 4 °C and 13,000 rpm for 15 min. The supernatant was transferred to a Eppendorf tube, and the precipitate was re-extracted in the same manner, combining the two extracted supernatants together. Then, supernatants were transferred into 1.5 mL of Eppendorf tubes, respectively, and then dried using a SpeedVac vacuum concentrator (Eppendorf, Germany) and nitrogen blow-down evaporator.

Preparation of colonic contents samples: The dry extracts of colonic contents samples were reconstituted in 100 μL methanol-water (4:1, *v/v*) containing the two IS (1 μg/mL of each IS), vortexed for 2 min, and centrifuged at 4 °C/13,000 rpm, for 10 min. Finally, 80 μL supernatant was transferred to autosampler vials as a test sample. Besides, 8 μL from each test sample were thoroughly mixed to create a pooled colonic contents sample, used as a quality control (QC) sample. For the QC sample, the analytical method was the same as the test samples, and the QC sample was inserted for every ten test samples to check the stability and performance of the instrument.

**Supplemental File 4. The UHPLC-Q-Orbitrap/MS method for the untargeted metabolomics analysis**

Using Thermo-Fisher Dionex UltiMate 3000 UHPLC-Q Exactive Orbitrap-MS and Xcalibur workstation (Thermo Fisher, USA) to acquire UHPLC-MS/MS raw data. The chromatographic separation of prefrontal cortex tissue samples was performed on an Acquity UPLC HSS T_3_ column. The mobile phase consisted of solvents A: 0.1% formic acid in water (v/v), and solvents B: acetonitrile, which programmed as follows: 0 ~ 0.5 min, 1% B; 0.5 ~ 3.5 min, 1% B - 53% B; 3.5 ~ 7.5 min, 53% B - 70% B; 7.5 ~ 9 min, 70% B - 90% B; 9 ~ 13 min, 90% B; 13 ~ 14 min, 90% - 1% B; 14 ~ 16 min, 1% B. The flow rate was set at 0.2 mL/min, and the injection volume was 5 μL. Mass spectrometry conditions: prefrontal cortex tissues samples were analyzed under positive and negative ionization modes via heated electrospray ionization (HESI) source; the scan mode was “Full Scan 35000 FWHM/dd-MS_2_” (Resolution 17500, NCE 25, Stepped NCE 50%); the spray voltage was set to 3.6 kV for the positive mode and 2.5 kV for the negative mode; capillary temperature, 320 °C; sheath gas flow, 35 arbitrary units; aux gas flow, 10 arbitrary units; scan range, *m/z* 60-1200.

**Supplemental File 5. The detailed processes of the metabolomics data processing parameters in Compound Discoverer 3.0 software**

The metabolomics data were analyzed by UPLC-MS/MS, and the UPLC-MS/MS raw data were imported to Compound Discoverer 3.0 to obtain the matched peak data. The metabolomics data processing parameters of Compound Discoverer 3.0 software were set as follows: mass range: 60-1200 Da; mass tolerance, 10 ppm; RT tolerance, 0.05 min; assignment threshold, 60; S/N Threshold, 5; intensity tolerance, 25%. The retention time, molecular formula, molecular weight, and peak area information of all data obtained from Compound Discoverer 3.0 software were imported to Microsoft Excel 2020, and the peak areas data were normalized by IS (yohimbine for negative ion mode, reserpine for positive ion mode).

**Supplemental File 6. Targeted Metabolomic Analysis for indole-3-acetic acid**

*6.1 Liquid Chromatography*

Indole-3-acetic acid in the prefrontal cortex and colon content samples were detected by UHPLC system (Agilent 1290 series, Agilent Technologies, CA, USA) combined with Q-TRAP 6500/MS (AB SCIEX, Massachusetts, USA) (UHPLC-Q-TRAP 6500/MS) in a dynamic multiple reaction monitoring (MRM) scan-mode. Chromatographic separation was conducted on an Agilent 1290 series UPLC system (Agilent Technologies, CA, USA) with an Acquity UPLC HSS T_3_ column (Waters, USA, 2.1 mm × 100 mm, 1.8 μm). The optimum separation was obtained under gradient elution with phase A (0.1% v/v formic acid in water) and phase B (acetonitrile). the gradient elution was carried out as: 0 ~ 0.5 min, 1% B; 0.5 ~ 3.5 min, 1% B - 53% B; 3.5 ~ 7.5 min, 53% B - 70% B; 7.5 ~ 9 min, 70% B - 90% B; 9 ~ 13 min, 90% B; 13 ~ 14 min, 90% - 1% B; 14 ~ 16 min, 1% B. The flow rate was 0.2 mL/min, the injected volume was 5 μL, and the column temperature was maintained at 35 °C.

*6.2 Mass Spectrometry*

AB SCIEX Q-TRAP 6500/MS (AB SCIEX, Massachusetts, USA) was equipped with an electronic spray ionization (ESI) source and directly connected with the outlet of UPLC. For the ion source parameters, the source temperature was set at 450℃; curtain gas (CUR) and two source gases (GS1 and GS2) were maintained at 20, 35, and 35 psi, respectively. The sprayer voltage was fixed at 5500 V for positive polarity, and the sprayer voltage was fixed at -5500 V for negative polarity. Multiple reaction monitoring (MRM) mode was used to acquire the quantitative data. The compound-dependent parameters, including MRM ion pairs, CE, and DP, are listed in Supplemental Table S1.

**Supplemental Figures:**


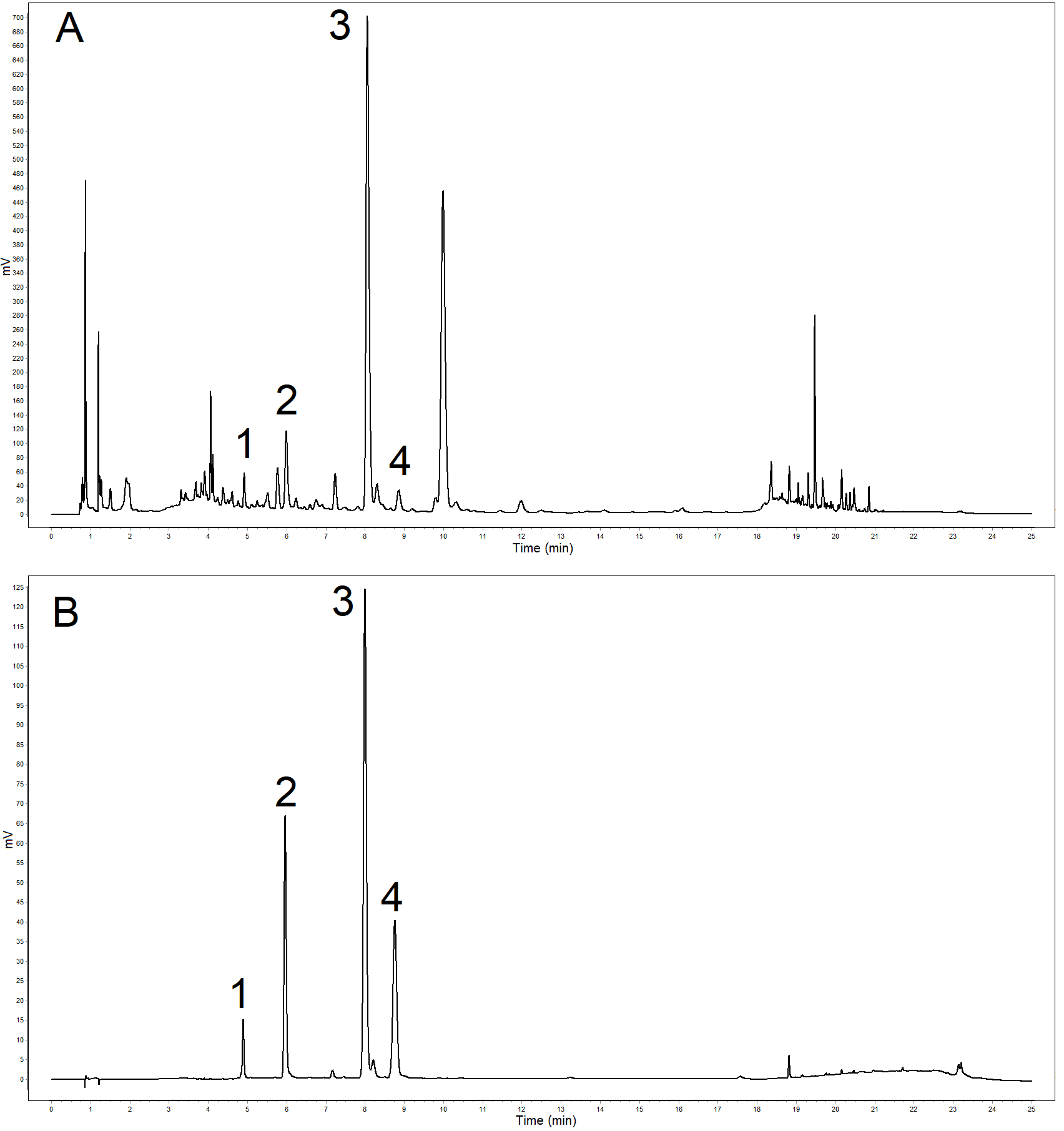


**Supplementary Fig. S1.** Chromatograms of Si-Ni-San (SNS) (A) and mixed standards (1: paeoniflorin, 2: liquiritin, 3: naringin,4: hesperidin) (B)


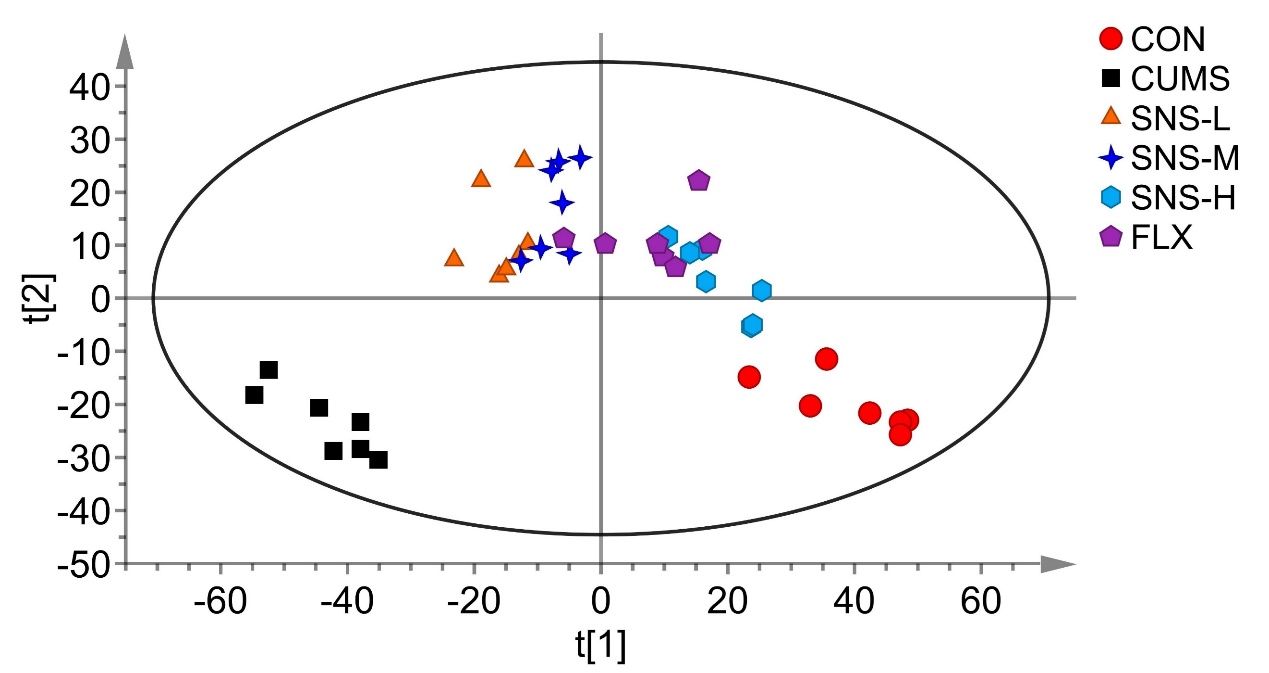


**Supplementary Fig. S2.** PLS-DA score plots from the CON group, CUMS group, SNS-L group, SNS-M group, SNS-H group, and FLX (*n* = 7).


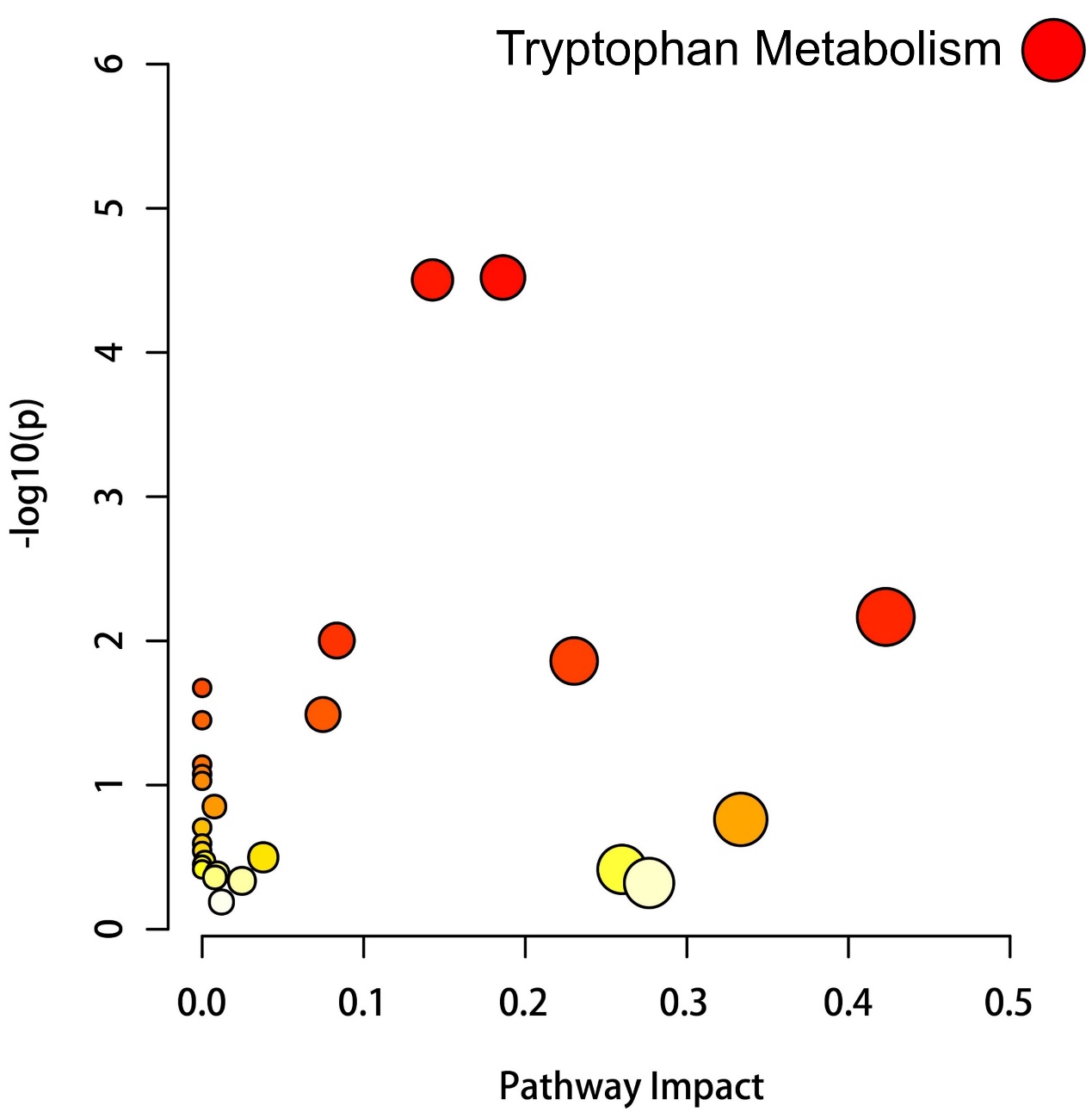


**Supplementary Fig. S3.** MetaboAnalyst pathway enrichment analysis of 33 overlapping differential metabolites.


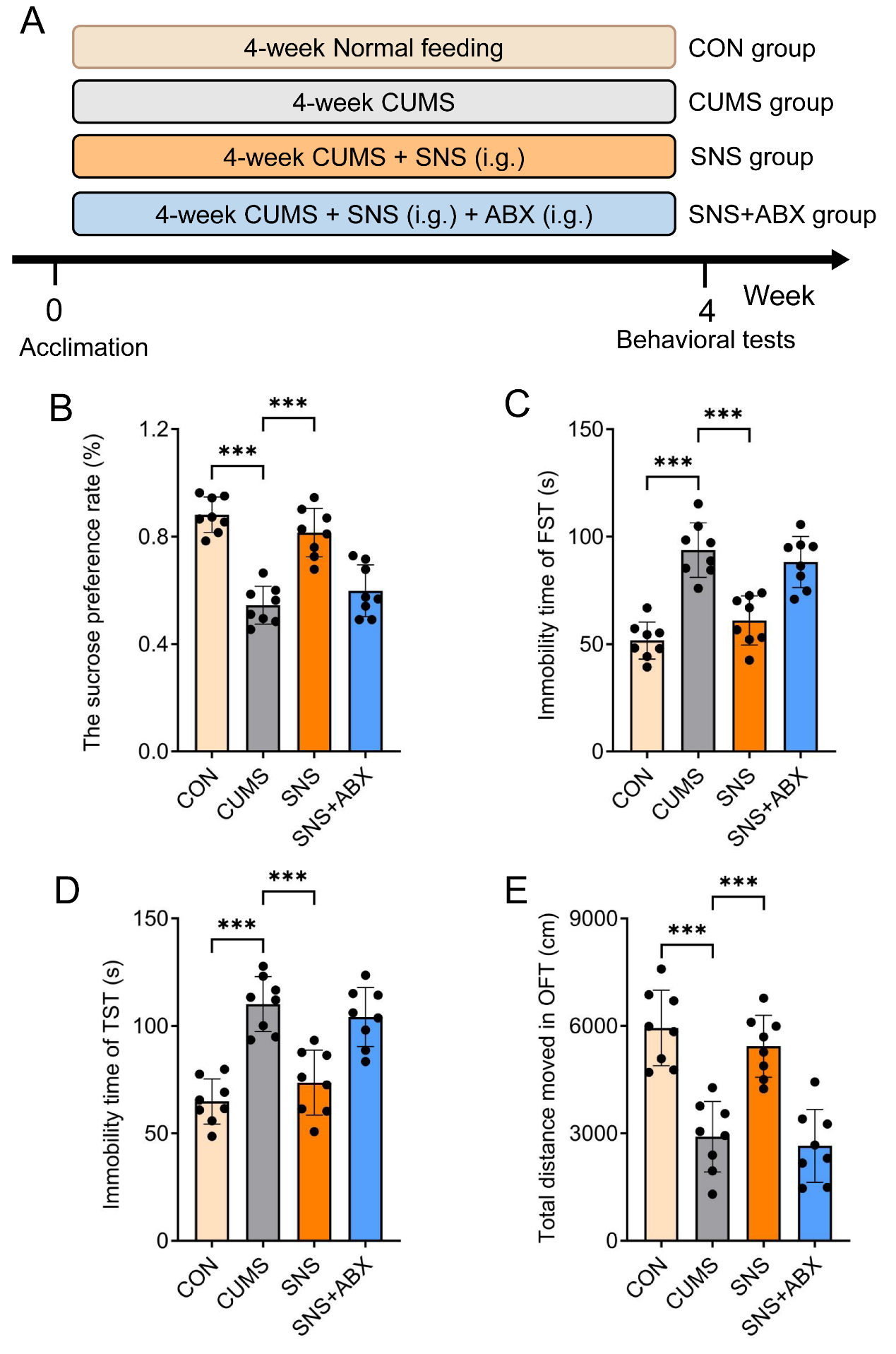


**Supplementary Fig. S4.** Gut microbiota depletion attenuates the antidepressant effects of SNS in CUMS mice. (A) Timeline of experimental procedures. (B) Sucrose preference rate in the SPT. (C) Immobility time recorded in the FST. (D) Immobility time recorded in the TST. (E) Total movement distance in the OFT. All results are shown as mean ± SD (*n* = 8). *P* values were computed via two-tailed Student’s t-test (between two groups) or one-way ANOVA (among several groups). ^***^*P* < 0.001, vs CUMS group.


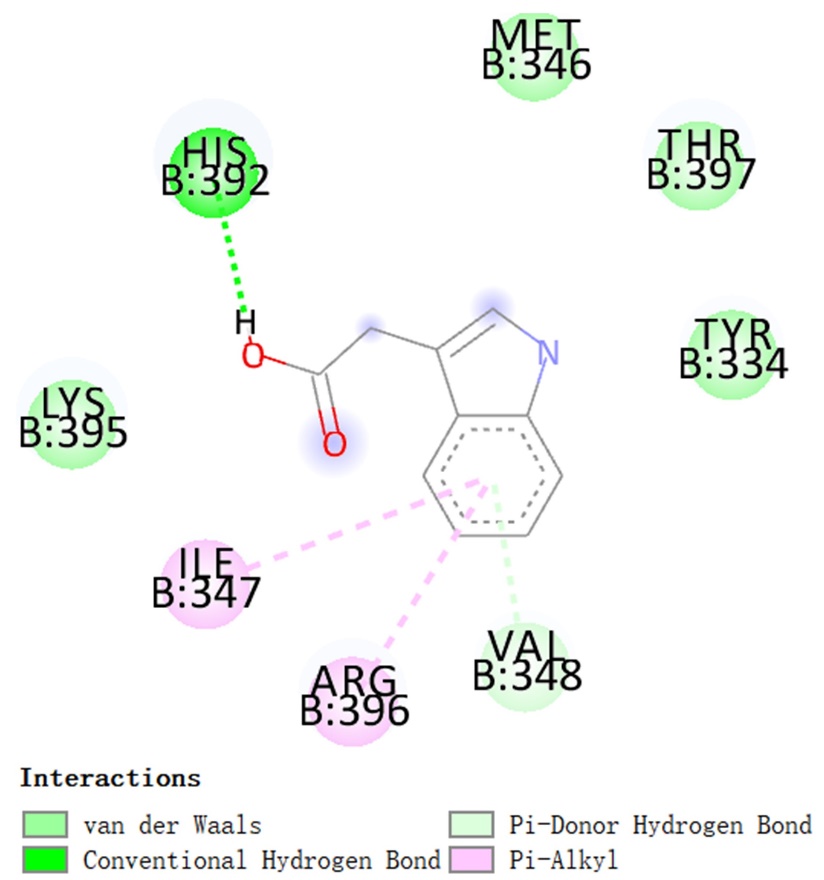


**Supplementary Fig. S5.** Analysis of the Three-Dimensional Binding Sites between AhR and IAA by Molecular Docking.


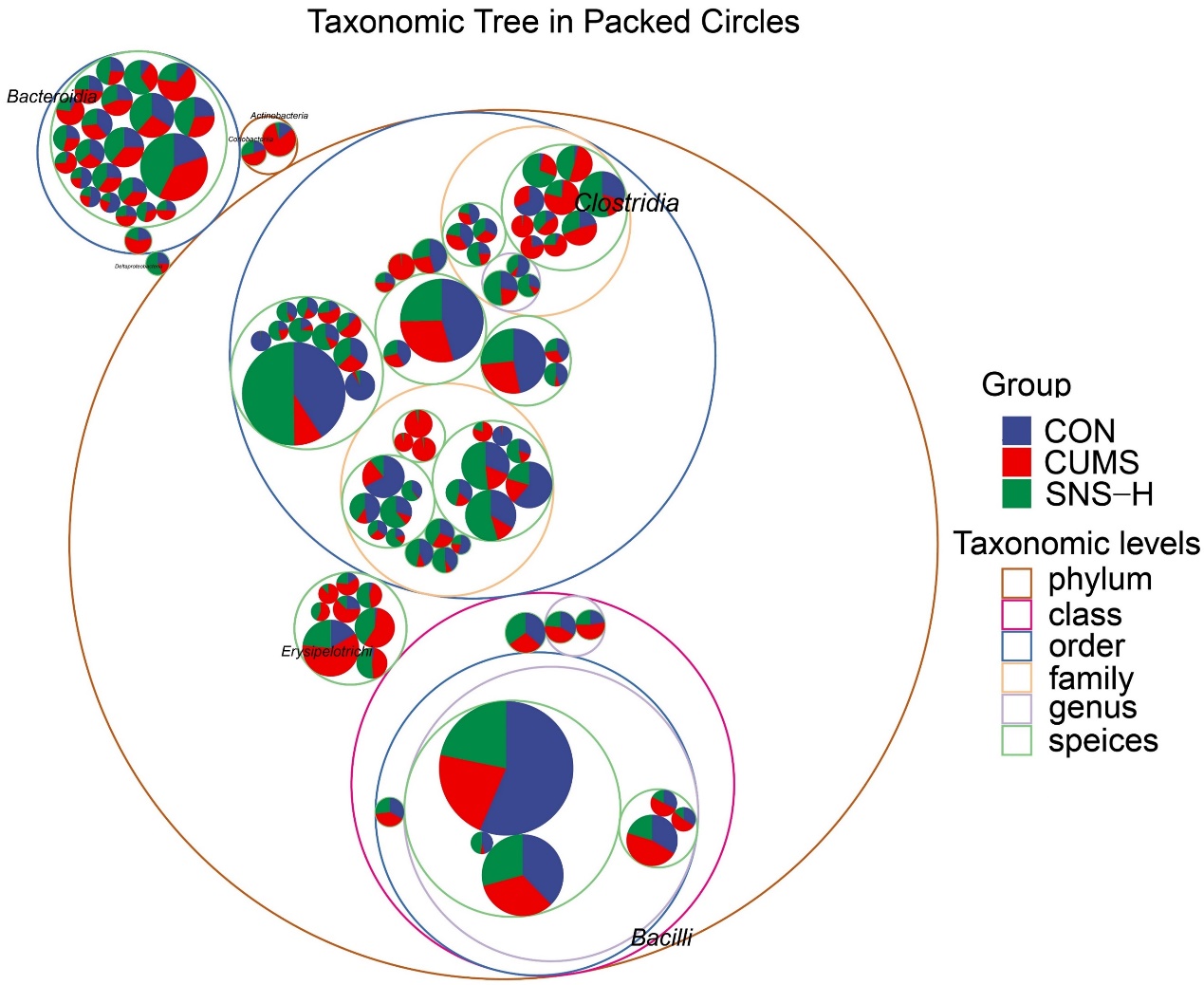


**Supplementary Fig. S6.** Taxonomic tree in packed circles. Taxonomic differences are based on 16S rRNA gene sequences extracted from the metagenome. The largest circles represent the phylum level, and the inner circles represent class, family, genus, and species.


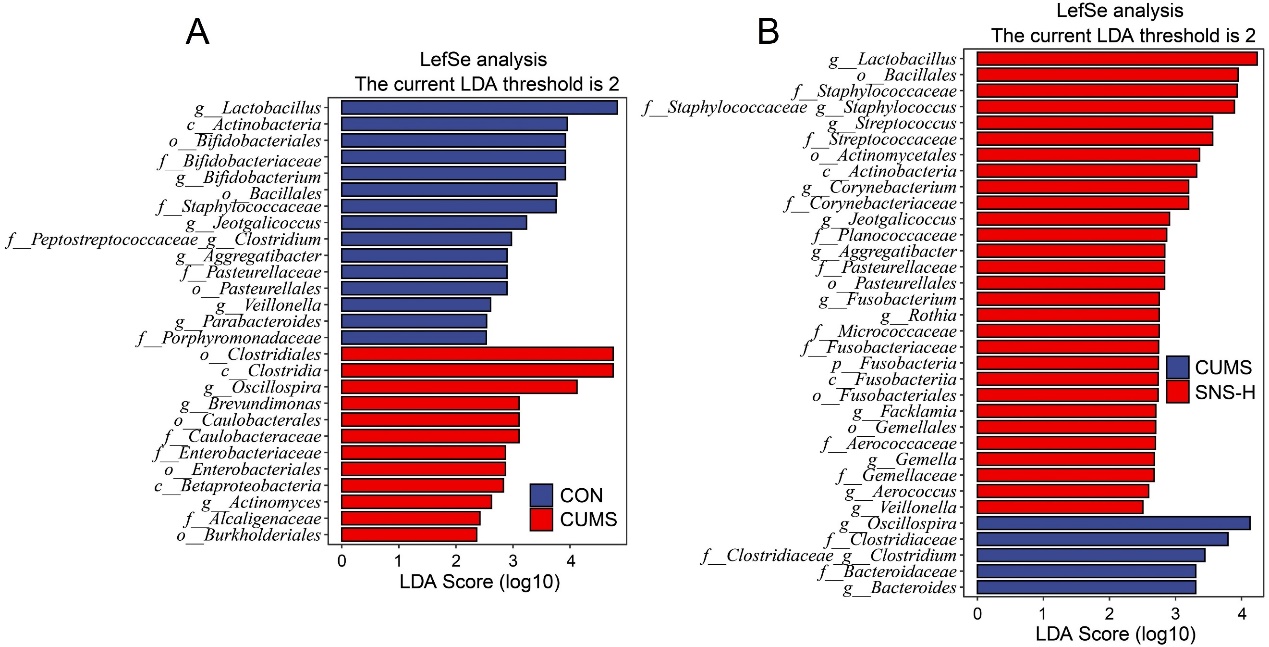


**Supplementary Fig. S7.** The LEfSe analysis was performed based on the linear discriminant analysis (LDA) To screen the differential bacteria among the CON, CUMS, and SNS-H groups (*P* < 0.05 and LDA > 2).


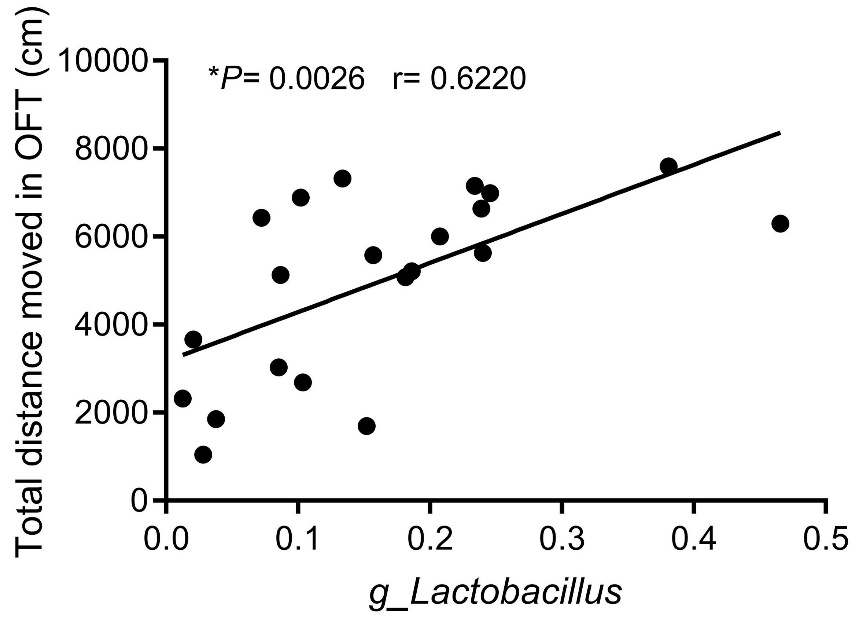


**Supplementary Fig. S8.** Correlation analysis between *lactobacillus* and total distance moved in OFT according to Pearson correlation coefficient.

**Supplemental Tables:**

**Supplementary Table S1**. MRM-based mass spectrometry parameters for the detection of indole-3-acetic acid

| Compounds | Formula | Q1/Q3 | DP (V) | CE (eV) | Ion mode |
| --- | --- | --- | --- | --- | --- |
| Indole-3-acetic acid | C_10_H_9_NO_2_ | 175.9-129.9 | 71 | 42 | [M+H]^+^ |
| Reserpine (IS) | C_33_H_40_N_2_O_9_ | 609.3-195.1 | 80 | 50 | [M+H]^+^ |
